# Thyroid dysfunction risk in systemic lupus erythematosus: a systematic review of case–control studies

**DOI:** 10.3389/fimmu.2026.1810304

**Published:** 2026-08-26

**Authors:** Rui-Cen Li, You-Yue Chen, An-Fang Huang, Wang-Dong Xu

**Affiliations:** 1 Health Management Center, West China Hospital, Sichuan University, Chengdu, Sichuan, China; 2 Department of Evidence-Based Medicine, School of Public health, Southwest Medical University, Luzhou, Sichuan, China; 3 Department of Rheumatology and Immunology, The Affiliated Hospital, Southwest Medical University, Luzhou, Sichuan, China

**Keywords:** case-control studies, risk factors, SLE, systematic review, thyroid dysfunction

## Abstract

**Objectives:**

This study investigated the prevalence of thyroid dysfunction—including clinical and subclinical hyperthyroidism, clinical and subclinical hypothyroidism, and euthyroid sick syndrome (ESS)—and its associated risk factors in patients with systemic lupus erythematosus (SLE) through case–control studies and meta-analysis.

**Materials and methods:**

The prevalence of thyroid dysfunction in SLE patients was first examined in two independent cohorts (Luzhou and Enshi). A meta-analysis was subsequently conducted using studies identified from PubMed and other databases to estimate the global prevalence of thyroid dysfunction among SLE patients. Factors associated with thyroid dysfunction in SLE were analyzed within the two cohorts, and meta-analysis was further employed to synthesize evidence on these associations.

**Results:**

The prevalence of all thyroid dysfunction subtypes was higher in SLE patients than in healthy controls. Meta-analysis confirmed that, at a global level, SLE patients had a higher prevalence of clinical hypothyroidism, subclinical hyperthyroidism, and ESS compared with healthy controls. Meta-analysis also identified significant associations between clinical hypothyroidism and renal involvement (*P* = 0.017) as well as low FT3 levels (*P* < 0.001). ESS was significantly associated with low C3 (*P* < 0.001) and low FT3 levels (*P* = 0.007).

**Conclusions:**

Thyroid dysfunction is prevalent among SLE patients worldwide. Severe renal involvement and decreased FT3 levels indicate an increased risk of clinical hypothyroidism in SLE, while reduced FT3 and C3 levels are associated with a higher risk of ESS.

## Introduction

Systemic lupus erythematosus (SLE) is a chronic autoimmune disease characterized by immune dysregulation and excessive production of autoantibodies (1, 2). Although its exact etiology remains unclear, current evidence suggests that genetic, infectious, and endocrine factors may contribute to disease development (3, 4). As the disease progresses, multiple organ systems can become involved (5, 6), leading to a range of complications including lupus nephritis (7) and neuropsychiatric manifestations such as lupus encephalopathy (8). In addition, SLE is frequently associated with thyroid dysfunction (9).

Thyroid dysfunction refers to the excessive or insufficient secretion of thyroid hormones produced by the thyroid gland, the largest endocrine organ in the body, resulting in altered metabolic activity (10). These hormones play essential roles in regulating metabolism (10) and supporting the development and maturation of the nervous system (11–13). Accumulating evidence suggests that the prevalence of thyroid dysfunction is significantly higher in patients with SLE than in the general population (14, 15). In the present study, we first conducted a case–control analysis to assess whether the prevalence of thyroid dysfunction is elevated in SLE patients. We then performed a meta-analysis to estimate the global prevalence of thyroid dysfunction among individuals with SLE. Additionally, we explored factors associated with thyroid dysfunction in this population.

## Methods

### Case–control study

#### Patients and healthy controls

Two independent cohorts were included to assess the prevalence of thyroid dysfunction in patients with SLE. The Luzhou cohort enrolled 635 SLE patients between 2018 and 2025, and the Enshi cohort enrolled 378 SLE patients during the same period. All patients met the 2019 EULAR/ACR classification criteria for SLE. For each cohort, an equal number of age- and sex-matched healthy controls (635 and 378, respectively) were recruited. Based on thyroid hormone values (FT3, FT4, and TSH), the patients were categorized into the following groups according to the American Thyroid Association (ATA) guidelines (16): (1) clinical hyperthyroidism: elevated FT4 with suppressed TSH, (2) subclinical hyperthyroidism: normal FT4 with suppressed TSH, (3) clinical hypothyroidism: low FT4 with elevated TSH, (4) subclinical hypothyroidism: normal FT4 with elevated TSH, and (5) Euthyroid sick syndrome (ESS): low FT4 or low FT3 with normal TSH. The reference ranges for FT4, FT3, and TSH are 12.0–22.0 pmol/L, 3.1–6.8 pmol/L, and 0.27–4.20 µIU/ml, respectively (17). All participants provided written informed consent. The study was approved by the Ethics Committee of the Affiliated Hospital of Southwest Medical University (approval no. KY2020180) and conducted in accordance with the Declaration of Helsinki. A clinical trial registration number was not applicable.

#### Demographic variables, clinical and laboratory indicators

Data related to demographic characteristics, including age and sex as well as clinical and laboratory parameters (e.g., white blood cell count (WBC), neutrophil count (NEU), and lymphocyte count (LYM)), were obtained from the electronic medical record systems of both participating hospitals for all SLE patients and healthy controls. Data on thyroid function indicators, including thyroid-stimulating hormone (TSH), free triiodothyronine (FT3), and free thyroxine (FT4), were also collected for both cohorts.

#### Prevalence of thyroid dysfunction and associated factors in SLE patients: analysis of two independent cohorts

The prevalence of each thyroid dysfunction subtype, including clinical and subclinical hyperthyroidism, clinical and subclinical hypothyroidism, and ESS, was compared between SLE patients and healthy controls in both the Luzhou and Enshi cohorts. Logistic regression analysis was performed separately in each cohort to identify factors associated with each subtype of thyroid dysfunction among patients with SLE.

### Meta-analysis

To comprehensively assess the global prevalence of thyroid dysfunction in patients with SLE and identify associated influencing factors, we conducted a meta-analysis integrating findings from our two cohorts with those of published studies. The systematic review and meta-analysis were performed in accordance with the Preferred Reporting Items for Systematic Reviews and Meta-Analysis (PRISMA) statement. The protocol was registered with PROSPERO (registration number: CRD42024553760).

#### Retrieval strategies

We systematically searched the following electronic databases from inception to December 31, 2025: Web of Science, PubMed, Medline, Embase, Cochrane Library, Scopus, China National Knowledge Infrastructure (CNKI), and China Biomedical Database (CBM). The search strategy combined key terms related to SLE (e.g., “systemic lupus erythematosus,” “SLE”) and thyroid dysfunction (e.g., “thyroid disease,” “hypothyroidism,” “hyperthyroidism”). Studies were included if they met the following criteria: (1) case–control or cohort study design, (2) thyroid dysfunction clearly defined according to established diagnostic criteria, (3) SLE diagnosis based on recognized classification criteria, and (4) odds ratios (ORs) with 95% confidence intervals (CIs) reported or sufficient data provided to calculate them. Studies were excluded if they met any of the following criteria: (1) case reports, reviews, conference abstracts, or letters, (2) duplicate publications, (3) non-clinical studies (e.g., animal experiments), (4) unavailable or incomplete data, (5) published in languages other than Chinese or English, or (6) otherwise failing to meet the inclusion criteria.

#### Literature search and data extraction

Two reviewers independently screened all retrieved records for eligibility. Disagreements were resolved through discussion or, if necessary, consultation with a third reviewer. Zotero was used for reference management and study selection. Studies were selected based on the predefined inclusion and exclusion criteria. For each eligible study, the following information was extracted: author(s), study region, year of publication, demographic characteristics of case and control groups, as well as relevant clinical findings and laboratory parameters.

### Study quality assessment

All studies included in this meta-analysis were case–control or cohort designs. Methodological quality was independently assessed by two reviewers using the Newcastle–Ottawa Scale (NOS). Any discrepancies were resolved through discussion.

### Statistical analysis

For the case–control studies, categorical variables were presented as frequencies and percentages and continuous variables as mean ± standard deviation or median with interquartile range, as appropriate. Normality of continuous variables was assessed using the Shapiro–Wilk test in both the Luzhou and Enshi cohorts. Associations between SLE and thyroid dysfunction subtypes were evaluated using chi-square test or Fisher’s exact test, as applicable. All tests were two-tailed, with the significance level set at *α* = 0.05. Statistical analysis was performed using R software (version 4.3.1).

For meta-analysis, it was conducted using R software to pool effect sizes and estimate the global prevalence of each thyroid dysfunction subtype, including clinical and subclinical hyperthyroidism, clinical and subclinical hypothyroidism, and ESS. Pooled prevalence with 95% confidence intervals were calculated. Between-study heterogeneity was assessed using the *I*^2^ statistic. A random-effects model was applied when *I*^2^ >50%; otherwise, a fixed-effects model was used. Forest plots were generated to visually summarize study-specific and pooled estimates. Sensitivity analysis was performed using the leave-one-out method. Publication bias was evaluated by visual inspection of funnel plots.

## Results

### Higher prevalence of thyroid dysfunction in SLE patients across two cohorts

#### Basic information in the Luzhou and Enshi cohorts

This study included two independent cohorts: Luzhou and Enshi. Owing to missing data, such as medications, disease duration, body mass index (BMI), SLEDAI score, and clinical features (lupus nephritis, lupus headache, arthritis, myositis, and rash) in both cohorts, the Luzhou cohort comprised 635 patients with SLE and 635 age- and sex-matched healthy controls for analysis. Among SLE patients, 88.50% were female, and the age was 43.50 ± 19.12 years, compared with 42.52 ± 13.55 years in the control group. The Enshi cohort included 378 SLE patients and 378 age- and sex-matched healthy controls for analysis. The proportion of female patients was 84.92%, with the age of 42.55 ± 14.31 years in the SLE group and 43.12 ± 13.65 years in the control group. Detailed laboratory and clinical characteristics for both cohorts are presented in **Supplementary Table 1**.

#### Analysis of prevalence of thyroid dysfunction in both cohorts

The prevalence of thyroid dysfunction subtypes among SLE patients and healthy controls in both the Luzhou and Enshi cohorts is summarized in **Table 1**. In the Luzhou cohort, patients with SLE exhibited a significantly higher prevalence of clinical hyperthyroidism, clinical hypothyroidism, subclinical hypothyroidism, and ESS compared with healthy controls (all *P* < 0.05). In the Enshi cohort, SLE patients showed a significantly higher prevalence of subclinical hyperthyroidism and ESS relative to controls (both *P* < 0.05).

**Table 1:** Distribution of thyroid dysfunction in Luzhou and Enshi cohorts.

Types of thyroid dysfunction	Luzhou cohort			Enshi cohort		
	SLE (*n*)	HC (*n*)	*P*-value	SLE (*n*)	HC (*n*)	*P*-value
Clinical hyperthyroidism	12	3	0.019	3	7	0.203
Subclinical hyperthyroidism	18	11	0.189	21	7	0.007
Clinical hypothyroidism	46	11	0.013	16	14	0.709
Subclinical hypothyroidism	83	49	0.002	69	59	0.332
ESS	31	0	<0.001	20	2	<0.001

SLE, systemic lupus erythematosus; HC, healthy control; ESS, euthyroid sick syndrome.

#### Prevalence of thyroid dysfunction in SLE: A global meta-analysis

**Figure 1:**
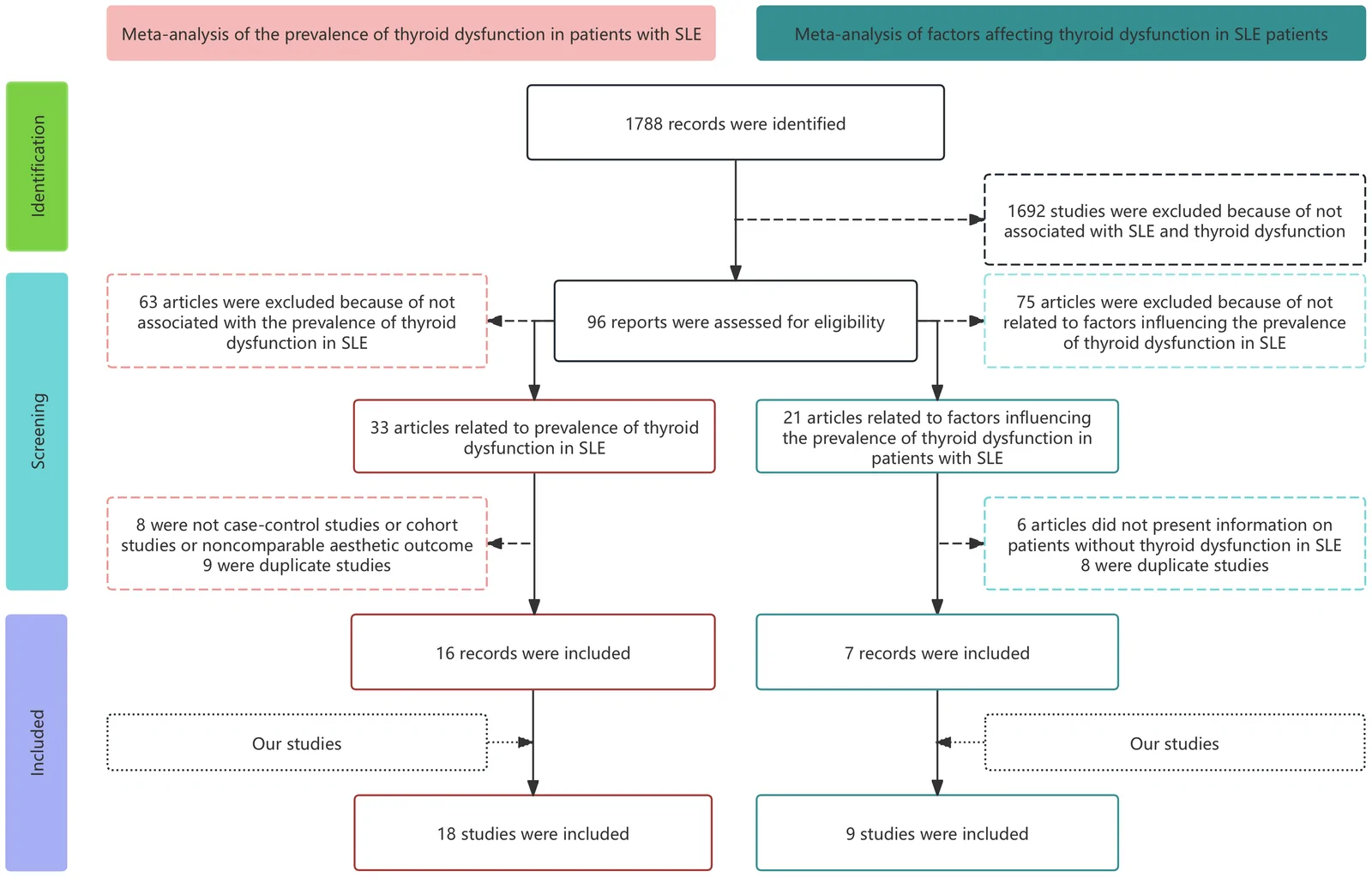
Flowchart of the meta-analysis discussing the prevalence and influencing factors of thyroid dysfunction in patients with SLE.

A total of 1,788 records were identified through systematic searches of Web of Science, PubMed, Medline, Embase, Cochrane Library, Scopus, CNKI, and CBM. After excluding 1,699 records unrelated to both SLE and thyroid dysfunction, 96 potentially eligible studies were retained. For the meta-analysis of thyroid dysfunction prevalence in SLE, an additional 63 studies not reporting prevalence data, with eight studies that were not case–control or cohort designs or lacked relevant outcome measures, were excluded, resulting in 16 eligible studies. The Luzhou and Enshi cohorts were also included, yielding a total of 18 studies for meta-analysis. The study selection process is illustrated in **Figure 1**. Methodological quality of the 18 included studies was assessed using the NOS. Studies with NOS scores ≥4 were considered moderate to high quality; no low-quality studies were identified (**Supplementary Table 2**). For each included study, the following information was extracted: study region, year of publication, thyroid dysfunction subtype(s) assessed, sample sizes of case and control groups, sex distribution, and age characteristics (**Table 2**) (9, 14, 15, 17–32).

**Table 2:** Information about the included studies for meta-analysis of the prevalence of thyroid dysfunction in patients with SLE.

Study	Region	Year	Thyroid dysfunction type	SLE (*n*)	Control (*n*)	Female, *n* (%)	Age (year)
Al-Awadhi, et al. (27)	Kuwait	2008	Clinical hyperthyroidism Clinical hypothyroidism Subclinical hypothyroidism ESS	60	577	SLE: 59 (98.33%) Control: 377 (65.34%)	SLE: 42.38 ± 10.80 Control: 36.88 ± 10.30
Domingues, et al. (18)	Brazil	2017	Clinical hyperthyroidism Subclinical hyperthyroidism Clinical hypothyroidism	79	159	SLE: 72 (91.14%) Control: 145 (91.19%)	SLE: 35.50 ± 12.10 Control: 35.70 ± 12.20
Watad, et al. (26)	Israel	2016	Clinical hyperthyroidism	5,018	25,090	SLE: 4,113 (81.96%) Control: 20,565 (81.96%)	SLE: 50.20 ± 17.40 Control: 50.20 ± 17.40
Watad, et al. (9)	Israel	2016	Clinical hypothyroidism	5,018	25,090	SLE: 4,113 (81.96%) Control: 20,565 (81.96%)	SLE: 50.22 ± 17.38 Control: 50.22 ± 17.38
Appenzeller, et al. (19)	Brazil	2009	Clinical hyperthyroidism Subclinical hyperthyroidism Clinical hypothyroidism Subclinical hypothyroidism	524	50	Control: 50 (100.00%)	SLE: 28.80 ± 11.00 Control: 32.30 ± 11.00
Antonelli, et al. (15)	Italy	2010	Subclinical hyperthyroidism Clinical hypothyroidism Subclinical hypothyroidism	213	426	SLE: 201 (94.37%) Control: 402 (94.37%)	SLE: 42.00 ± 15.00 Control: 39.00 ± 14.00
Liu, et al. (28)	Taiwan	2019	Clinical hyperthyroidism Clinical hypothyroidism	2,796	11,184	SLE: 2,483 (88.81%) Control: 9,932 (88.81%)	SLE: 33.40 ± 14.90 Control: 32.40 ± 14.90
Lin, et al. (29)	Taiwan	2015	Clinical hyperthyroidism Clinical hypothyroidism	1,633	6,532	SLE: 1,464 (89.65%) Control: 5,856 (89.65%)	SLE: 34.90 ± 14.80 Control: 35.20 ± 15.00
Kumar, et al. (20)	India	2012	Clinical hyperthyroidism Subclinical hyperthyroidism Clinical hypothyroidism Subclinical hypothyroidism ESS	100	100	SLE: 88 (88.00%) Control: 88 (88.00%)	Total: 26.60 ± 9.42
Mader et al. (21)	Israel	2007	Subclinical hypothyroidism	77	52	SLE: 64 (83.12%) Control: 33 (63.46%)	SLE: 37.30 ± 11.70 Control: 34.20 ± 10.70
Li et al. (22)	China	2021	Clinical hyperthyroidism Subclinical hyperthyroidism Clinical hypothyroidism Subclinical hypothyroidism ESS	672	605	SLE: 599 (89.14%) Control: 544 (89.92%)	SLE: 33.00 (24.00, 44.00) HC: 35.00 (30.00, 39.00)
Posselt et al. (23)	Brazil	2017	Clinical hypothyroidism	301	141	SLE: 282 (93.6%) Control: 130 (92.1%)	SLE: 42.00 (31.00, 51.00) Control: 39.00 (28.50, 52.00)
Na-Nan et al. (14)	Thailand	2024	Subclinical hyperthyroidism Clinical hypothyroidism Subclinical hypothyroidism	100	100	SLE: 95 (95.00%) Control: 95 (95.00%)	SLE: 37.28 ± 11.92 Control: 37.24 ± 11.11
Zhang et al. (24)	China	2023	Clinical hyperthyroidism Subclinical hyperthyroidism Clinical hypothyroidism Subclinical hypothyroidism ESS	253	70	SLE: 209 (82.61%) Control: 46 (8.57%)	SLE: 14.00 (12.00, 16.00) Control: 13.00 (10.00, 13.00)
Ni et al. (17)	China	2022	Clinical hypothyroidism	856	856	SLE: 785 (91.71%) Control: 785 (91.71%)	SLE: 41.11 ± 14.28 Control: 42.29 ± 11.92
Athanassiou et al. (25)	Greece	2023	Clinical hyperthyroidism Clinical hypothyroidism Subclinical hypothyroidism	45	45	SLE: 41 (91.11%) Control: 41 (91.11%)	SLE: 47.97 ± 2.17
Luzhou cohort	China	2025	Clinical hyperthyroidism Subclinical hyperthyroidism Clinical hypothyroidism Subclinical hypothyroidism ESS	635	635	SLE: 562 (88.50%) Control: 560 (88.19%)	SLE: 43.50 ± 19.12 Control: 42.52 ± 13.55
Enshi cohort	China	2025	Clinical hyperthyroidism Subclinical hyperthyroidism Clinical hypothyroidism Subclinical hypothyroidism ESS	378	378	SLE: 321 (84.92%) Control: 318 (84.13%)	SLE: 42.55 ± 14.31 Control: 43.12 ± 13.65

SLE, systemic lupus erythematosus; ESS, euthyroid sick syndrome.

**Figure 2:**
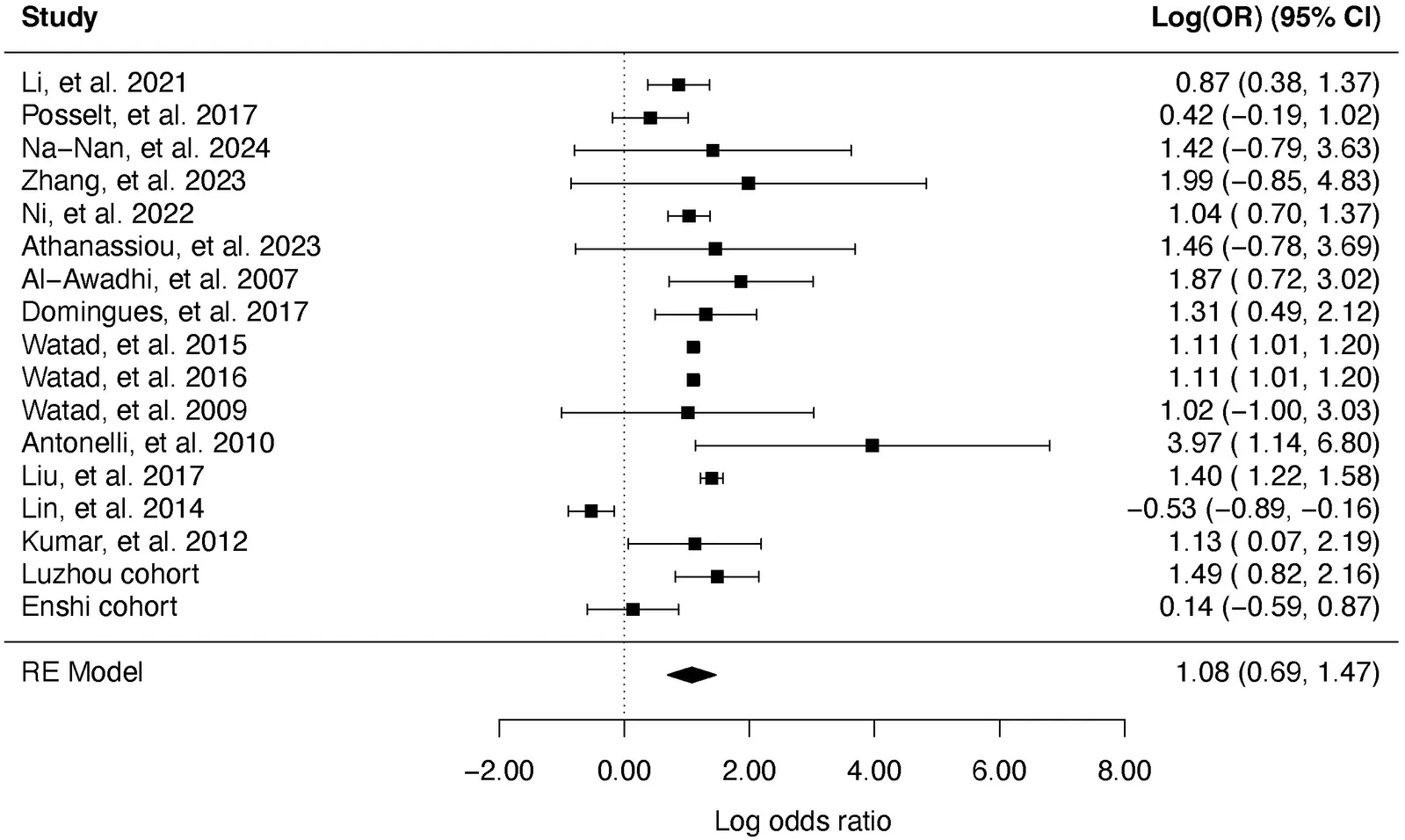
Forest plot of clinical hypothyroidism in patients with SLE. The boxes in the middle portion of this forest plot represent study-specific effect sizes for each study. The confidence interval for the effect size estimates for each study is represented by a horizontal line segment, which shows the range of uncertainty in the effect size, with shorter horizontal segments indicating higher study precision. The bottom box and line segment indicate the overall effect size of the meta-analysis and its confidence interval.

To visualize the global distribution of each thyroid dysfunction subtype, we generated world maps illustrating the prevalence of clinical hyperthyroidism, subclinical hyperthyroidism, clinical hypothyroidism, subclinical hypothyroidism, and ESS in patients with SLE (**Supplementary Figures 1**–**S5**). These maps revealed that thyroid dysfunction is common among SLE patients worldwide, with notable inter-country variability. We next performed meta-analysis to quantitatively assess whether the risk of each thyroid dysfunction subtype is elevated in patients with SLE. For clinical hypothyroidism, 17 studies were included. A random-effects model was applied due to heterogeneity, and the pooled results demonstrated a significantly increased prevalence of clinical hypothyroidism in SLE patients compared with controls (**Figure 2**). Meta-analysis of clinical hyperthyroidism, subclinical hyperthyroidism, subclinical hypothyroidism, and ESS was also conducted, with the results summarized in **Supplementary Figures 6**–**S9**. All four subtypes showed a consistently higher prevalence in SLE patients relative to healthy controls.

#### Factors associated with thyroid dysfunction in SLE: two-cohort analysis

Given the substantial global variation in thyroid dysfunction prevalence and its potential implications for disease management, we further investigated factors associated with each thyroid dysfunction subtype in SLE patients using data from the Luzhou and Enshi cohorts. Univariate and multivariate logistic regression analyses were performed for each subtype. In the Luzhou cohort, multivariate analysis revealed the following independent associations: clinical hyperthyroidism: mean corpuscular volume (MCV) (OR = 0.867, *P* < 0.001) and AST/ALT ratio (OR = 0.105, *P* = 0.007) were negatively associated, while thyroid peroxidase antibody (TPOAb) (OR = 1.004, *P* < 0.001) was positively associated; clinical hypothyroidism: chloride (Cl) (OR = 1.173, *P* = 0.003) and TSH (OR = 1.261, *P* < 0.001) were positively associated, whereas FT3 (OR = 0.258, *P* = 0.001) was negatively associated; subclinical hypothyroidism: age (OR = 1.018, *P* = 0.030) was a positive risk factor, while calcium (Ca) (OR = 0.148, *P* = 0.006) was negatively associated; and ESS: uric acid (UA) (OR = 1.003, *P* = 0.008) was positively associated, whereas FT3 (OR = 0.091, *P* < 0.001) and TSH (OR = 0.824, *P* = 0.024) were negatively associated (**Supplementary Tables 3**–**S11**).

We further examined factors associated with thyroid dysfunction among SLE patients in the Enshi cohort. Multivariate logistic regression analysis revealed the following independent associations: clinical hyperthyroidism: FT4 was positively associated with clinical hyperthyroidism in SLE patients (OR = 1.941, *P* = 0.013); subclinical hyperthyroidism: LYM emerged as a protective factor (OR = 0.368, *P* = 0.033), whereas red cell distribution width-standard deviation (RDW-SD) was identified as a risk factor (OR = 1.035, *P* = 0.049); clinical hypothyroidism: gamma-glutamyltransferase (GGT) and FT4 were negatively associated, while TSH was positively associated with clinical hypothyroidism; subclinical hypothyroidism: female sex (OR = 0.249, *P* = 0.039) and albumin-to-globulin ratio (A/G) (OR = 0.465, *P* = 0.039) were negatively associated, whereas TSH was positively associated (OR = 1.037, *P* = 0.023); and ESS: complement C3 (OR = 0.124, *P* = 0.049), FT3 (OR = 0.463, *P* = 0.008), and FT4 (OR = 0.639, *P* < 0.001) were negatively associated with ESS, while total bilirubin (TBIL) (OR = 1.066, *P* = 0.040) was identified as a risk factor. Detailed regression results for the Enshi cohort are provided in **Supplementary Tables 12**–**S21**.

#### Risk factors for thyroid dysfunction in SLE: a meta-analysis

We conducted a meta-analysis to identify factors associated with each thyroid dysfunction subtype in patients with SLE. Following the initial search, 1,692 records unrelated to both SLE and thyroid dysfunction were excluded. Of the remaining studies, 75 were excluded for not reporting factors associated with thyroid dysfunction in SLE. Among the 21 potentially eligible studies, six were excluded due to insufficient data on thyroid dysfunction outcomes, and eight were duplicates, leaving seven eligible studies. After including the two cohorts from the present study, a total of nine studies were included in the meta-analysis. The study characteristics, including author, region, year of publication, thyroid dysfunction subtype, and case and control sample sizes, are summarized in **Table 3** (9, 17, 19, 22, 26, 28, 33). Meta-analysis demonstrated that renal involvement was significantly associated with an increased risk of clinical hypothyroidism in SLE patients (OR = 2.761, *P* = 0.017), while the FT3 level was negatively associated with clinical hypothyroidism (OR = -0.547, *P* < 0.001). For ESS, both C3 (OR = -0.231, *P* < 0.001) and FT3 (OR = -1.263, *P* = 0.007) were negatively associated with ESS in SLE patients (**Supplementary Table 22**). Due to the insufficient number of studies, meta-analysis for other thyroid dysfunction subtypes could not be performed.

**Table 3:** Information about the included studies for meta-analysis of factors influencing the prevalence of thyroid dysfunction in patients with SLE.

Study	Region	Year	Thyroid dysfunction type	SLE with thyroid dysfunction (*n*)	SLE without thyroid dysfunction (*n*)
Watad et al. (26)	Israel	2015	Clinical hyperthyroidism	130	4,888
Watad et al. (9)	Israel	2016	Clinical hypothyroidism	782	4,236
Li et al. (22)	China	2021	Clinical hyperthyroidism	6	666
			Subclinical hyperthyroidism	12	660
			Clinical hypothyroidism	58	614
			Subclinical hypothyroidism	53	619
			ESS	333	339
Appenzeller et al. (19)	Brazil	2009	Clinical hypothyroidism	9	77
Gao et al. (33)	China	2011	Subclinical hypothyroidism	10	731
Liu et al. (28)	Taiwan	2019	Clinical hyperthyroidism	180	2,616
			Clinical hypothyroidism	237	2,559
Ni et al. (17)	China	2022	Clinical hypothyroidism	132	724
Luzhou cohort	China	2024	Clinical hyperthyroidism	12	622
			Subclinical hyperthyroidism	18	616
			Clinical hypothyroidism	46	588
			Subclinical hypothyroidism	83	551
			ESS	31	603
Enshi cohort	China	2024	Clinical hyperthyroidism	3	375
			Subclinical hyperthyroidism	21	357
			Clinical hypothyroidism	16	362
			Subclinical hypothyroidism	69	309
			ESS	20	358

SLE, systemic lupus erythematosus; ESS, euthyroid sick syndrome.

## Discussion

In the present case–control study, patients with SLE exhibited a significantly higher prevalence of all thyroid dysfunction subtypes compared with healthy controls across two independent cohorts. Meta-analysis further confirmed that, at the global level, the prevalence of clinical hypothyroidism, subclinical hyperthyroidism, and ESS was significantly elevated in individuals with SLE. These findings indicate that SLE patients worldwide face an increased risk of developing thyroid dysfunction, which may complicate disease prevention and clinical management. To further elucidate this association, we investigated factors related to specific thyroid dysfunction subtypes in both cohorts and performed meta-analysis to synthesize evidence on potential determinants. In the Luzhou cohort, MCV, AST/ALT ratio, and TPOAb were identified as potential correlates of clinical hyperthyroidism in SLE. In the Enshi cohort, elevated FT4 levels were significantly associated with an increased risk of clinical hyperthyroidism. Meta-analysis revealed that renal involvement and TSH levels were positively associated with clinical hypothyroidism, while higher FT3 levels emerged as a protective factor against clinical hypothyroidism in SLE. Collectively, our findings demonstrate that distinct subtypes of thyroid dysfunction in SLE are associated with partially different sets of clinical and laboratory factors. These results underscore the importance of subtype-specific risk assessment in clinical practice. Moving forward, clinicians should give greater attention to these differentially associated factors to facilitate early detection and personalized management of thyroid dysfunction in patients with SLE.

Our case–control study identified several clinical and laboratory indices, including MCV, RDW-SD, TPOAb, age, and sex, as factors significantly associated with thyroid dysfunction subtypes in patients with SLE. MCV was inversely associated with clinical hyperthyroidism in SLE. This relationship may be multifactorial. SLE is characterized by a sustained elevation of pro-inflammatory cytokines such as IFN-γ and IL-17 (34, 35), which are known to suppress erythropoiesis and reduce MCV (36, 37). These cytokines may also impair thyroid function through the disruption of TSH receptor signaling (38, 39). In addition, anemia and chronic malnutrition are common in SLE (40, 41), both of which may contribute to the development of clinical hyperthyroidism (41). RDW-SD was positively associated with subclinical hyperthyroidism. Chronic inflammation in SLE not only suppresses erythrocyte production but also promotes erythrocyte destruction (36, 37), leading to elevated RDW-SD. Concurrently, inflammatory cytokines may interfere with thyroid hormone synthesis (38, 39), thereby predisposing SLE patients to subclinical hyperthyroidism. Nutritional deficiencies frequently observed in SLE may further exacerbate these abnormalities (41). TPOAb was positively associated with clinical hypothyroidism in SLE patients, consistent with previous reports of elevated TPOAb levels in this population (25). TPOAb is known to mediate the autoimmune destruction of thyroid follicular cells, ultimately impairing hormone synthesis and leading to overt hypothyroidism (42, 43). Advanced age was identified as a risk factor for subclinical hypothyroidism in SLE, corroborating findings from population-based studies (44, 45). Age-related thyroid involution, including follicular atrophy and reduced iodine metabolism, may compound autoimmune-mediated thyroid damage in SLE patients (42, 43), thereby facilitating the onset of subclinical hypothyroidism (46). Female sex was associated with an increased risk of subclinical hypothyroidism in SLE, consistent with the higher prevalence of thyroid dysfunction reported in women (47, 48). Hyperprolactinemia, more frequently observed in female SLE patients, may stimulate thyroid antibody production and enhance the activity of thyroid-hormone-synthesizing enzymes such as TPO, paradoxically leading to reduced hormone output and promoting subclinical hypothyroidism.

Renal involvement was a significant risk factor for clinical hypothyroidism in patients with SLE. Several interrelated mechanisms may underlie this association. First, kidney damage, particularly lupus nephritis, a common complication of SLE (49), can lead to urinary loss of thyroid hormones and their binding proteins. Sustained hypothyroxinemia may eventually progress to overt hypothyroidism, manifesting as clinical hypothyroidism. Second, SLE is fundamentally characterized by immune dysregulation (50). Impaired regulatory T cell function and hyperactivation of helper T cells and B cells result in excessive autoantibody production (6, 51). Some of these autoantibodies may cross-react with thyroid follicular cells, promoting autoimmune destruction and impairing thyroid hormone synthesis. Third, chronic kidney disease and concomitant hypothyroidism may mutually reinforce each other. Reduced glomerular filtration rate, associated with both lupus nephritis and hypothyroidism, can decrease the renal clearance of thyroid hormones and their binding proteins (49, 52, 53), leading to hormone retention and further dysregulation of thyroid function. These mechanisms together suggest a bidirectional and multifactorial relationship between renal involvement and clinical hypothyroidism in SLE, with immune-mediated injury, urinary hormone loss, and impaired clearance all contributing to increased risk.

In our study, medication usage is missing. In fact, glucocorticoids exert complex, dual effects on thyroid function. They suppress the hypothalamic–pituitary–thyroid (HPT) axis primarily by inhibiting hypothalamic TRH and pituitary TSH secretion, leading to reduced thyroid stimulation. Additionally, they directly impair the peripheral conversion of T4 to T3 by inhibiting type 1 and type 2 deiodinases while also promoting inactive reverse T3 formation. This results in decreased FT3 levels independently of TSH. Clinically, this is relevant in critical illness (non-thyroidal illness syndrome) and during high-dose glucocorticoid therapy. However, the effect is dose and duration dependent. Chronic low-dose use may have minimal impact. Importantly, these changes are often adaptive, reducing metabolic demand. Clinicians should differentiate this drug-induced suppression from true central hypothyroidism, as recovery of the HPT axis typically follows glucocorticoid withdrawal. Monitoring FT3 alone can be misleading. Concurrent TSH, FT4, and clinical context are essential for an accurate interpretation. Cautious glucocorticoid tapering is recommended to avoid abrupt metabolic shifts. Overall, while glucocorticoids lower FT3, this effect is usually reversible and physiological, not necessarily indicating permanent thyroid damage. Proper management requires balancing anti-inflammatory benefits against potential thyroid axis modulation.

Our meta-analysis demonstrated that reduced FT3 levels are significantly associated with clinical hypothyroidism in patients with SLE. Low FT3 typically reflects diminished thyroid hormone secretion from the thyroid gland (54). In SLE, autoimmune-mediated injury to thyroid follicular cells may directly impair hormone synthesis, particularly FT3 (55). Notably, decreased FT3 also serves as a hallmark of low T3 syndrome, a physiological adaptation observed in critical illness and severe infection (56, 57). Patients with SLE may be particularly susceptible to this condition due to chronic systemic inflammation and immune dysregulation (58). Glucocorticoids, a mainstay of SLE treatment, can suppress the hypothalamic–pituitary–thyroid axis with long-term use, leading to reduced FT3 levels (59, 60). During active SLE, heightened autoimmune activity increases the risk of thyroid tissue destruction (61). Concurrently, systemic inflammation may disrupt thyroid hormone synthesis and peripheral conversion, exacerbating hypothyroidism (62). Finally, the shared genetic susceptibility between SLE and autoimmune thyroid disease may predispose certain individuals to develop both conditions concurrently (63). Therefore, these mechanisms suggest that low FT3 in SLE patients with clinical hypothyroidism is multifactorial, resulting from autoimmune thyroid injury, inflammation, treatment effects, and potentially overlapping genetic risk.

Low C3 level was a significant negative correlate of ESS in patients with SLE. Given the central role of complement in SLE pathogenesis, this association may reflect several interconnected mechanisms. First, hypocomplementemia in SLE indicates heightened immune activation and consumption, which exacerbates underlying immune dysregulation (64). This state of heightened autoimmune activity may increase susceptibility to ESS. Second, inflammatory cytokines can disrupt the hypothalamic–pituitary–thyroid (HPT) axis, impairing central regulation of thyroid hormone secretion and contributing to the characteristic low T3 state of ESS (65). Third, SLE is frequently accompanied by adaptive endocrine changes aimed at reducing energy expenditure and preserving vital organ function. These physiological adjustments may themselves perturb thyroid hormone homeostasis, and pre-existing complement deficiency may amplify such disturbances. Finally, SLE is often complicated by severe systemic conditions, including infections and renal insufficiency (66, 67), that provoke excessive inflammatory responses. These intercurrent illnesses can suppress HPT axis’ function and directly impair thyroid hormone synthesis, particularly FT3, thereby predisposing SLE patients to ESS. Low C3 levels, as a marker of both disease activity and complement consumption, may serve as a sentinel indicator of vulnerability to this pathway. Taken together, these mechanisms suggest that low C3 reflects an underlying state of immune activation, inflammation, and clinical vulnerability that collectively increases the risk of ESS in patients with SLE.

Several limitations of this study should be acknowledged. First, in the case–control component, data were retrospectively collected from electronic medical records, and a number of cases were excluded due to missing variables. As such, additional factors such as medication usage, disease duration, BMI, SLEDAI score, and clinical features (lupus nephritis, lupus headache, arthritis, myositis, and rash) potentially contributing to thyroid dysfunction in SLE may not have been captured and warrant further investigation in future studies. Similarly, most of the included studies missed data about medication usage, disease duration, BMI, SLEDAI score, and clinical features (lupus nephritis, lupus headache, arthritis, myositis, rash), resulting in no ability to conduct the meta-analysis that discussed the association of medications, SLEDAI score, disease duration, and BMI with SLE patients with thyroid dysfunction. Second, in the meta-analysis, we generated world maps to visualize the global distribution of thyroid dysfunction prevalence among SLE patients. However, due to the limited number of available studies from certain regions, the current maps could not fully represent the epidemiological landscape across all populations. Future research incorporating large-scale, multicenter studies is needed to provide a more comprehensive and geographically diverse assessment.

In conclusion, our case–control study revealed a higher prevalence of thyroid dysfunction in SLE patients than in healthy controls, a finding corroborated by meta-analysis. Renal involvement and low FT3 levels were associated with clinical hypothyroidism, while reduced C3 and FT3 were linked to ESS. This systematic evaluation of global epidemiological patterns provides a basis for future clinical monitoring of endocrine abnormalities in SLE.

## Data Availability

The raw data supporting the conclusions of this article will be made available by the authors, without undue reservation.

## Supplementary material

Supplementary Figure 1 World prevalence map of clinical hyperthyroidism in patients with SLE. This map visualizes the prevalence of clinical hyperthyroidism among SLE patients in countries around the world.

Supplementary Figure 2 World prevalence map of subclinical hyperthyroidism in patients with SLE. This map visualizes the prevalence of subclinical hyperthyroidism among SLE patients in countries around the world.

Supplementary Figure 3 World prevalence map of clinical hypothyroidism in patients with SLE. This map visualizes the prevalence of clinical hypothyroidism among SLE patients in countries around the world.

Supplementary Figure 4 World prevalence map of subclinical hypothyroidism in patients with SLE. This map visualizes the prevalence of subclinical hypothyroidism among SLE patients in countries around the world.

Supplementary Figure 5 World prevalence map of ESS in patients with SLE. This map visualizes the prevalence of ESS among SLE patients in countries around the world.

Supplementary Figure 6 Forest plot of clinical hyperthyroidism in patients with SLE. A total of six eligible studies were included in this part of the meta-analysis, in which the total effect size and its confidence interval was -0.13 (-1.22, 0.96).

Supplementary Figure 7 Forest plot of subclinical hyperthyroidism in patients with SLE. For the meta-analysis of subclinical hyperthyroidism, five studies were included, and the total effect size and its confidence interval was 0.84 (0.35, 1.33). The square at the bottom of the figure indicates the effect size is located to the right of the null line.

Supplementary Figure 8 Forest plot of subclinical hypothyroidism in patients with SLE. In the meta-analysis of the prevalence of subclinical hypothyroidism, the log (OR) and its 95% CI was 1.29 (-0.00, 2.58), which overlapped with the null line.

Supplementary Figure 9 Forest plot of ESS in patients with SLE. A total of five studies were included. The total effect size and its 95% CI was 3.61 (2.98, 4.24), which is located to the right of the null line.
