# Supplementary material for: Thyroid dysfunction risk in systemic lupus erythematosus: a systematic review of case–control studies
Source: Front Immunol. 2026 Aug 26;17:1810304. doi: 10.3389/fimmu.2026.1810304 (PMC13553969; doi:10.3389/fimmu.2026.1810304)
Supplement: Supplementary file 1 [file Table1.docx]

Supplementary table 1 Characteristics of the population in the Luzhou and Enshi cohorts.

| Variables | Luzhou Cohort | | |  | Enshi Cohort | | |
| --- | --- | --- | --- | --- | --- | --- | --- |
|  | SLE | HC | P value |  | SLE | HC | P value |
| WBC-Blood (10^9^/L) | 5.405 (3.743, 8.145) | 5.650 (4.830, 6.625) | 0.004 |  | 5.970 (4.505, 8.585) | 5.187 (4.540, 6.148) | <0.001 |
| NEU-Blood (10^9^/L) | 3.915 (2.442, 6.188) | 3.317 (2.732, 4.028) | <0.001 |  | 4.161 (0.845, 1.639) | 2.970 (2.502, 3.675) | <0.001 |
| LYM (10^9^/L) | 1.010 (0.662, 1.480) | 1.725 (1.400, 2.060) | <0.001 |  | 1.250 (0.845, 1.639) | 1.660 (1.470, 1.950) | <0.001 |
| MONO (10^9^/L) | 0.360 (0.210, 0.510) | 0.300 (0.250, 0.370) | 0.123 |  | 0.470 (0.350, 0.608) | 0.320 (0.280, 0.387) | <0.001 |
| EOS (10^9^/L) | 0.030 (0.010, 0.080) | 0.100 (0.060, 0.160) | 0.489 |  | 0.030 (0.010, 0.080) | 0.390 (0.180, 0.600) | <0.001 |
| BASO (10^9^/L) | 0.010 (0.010, 0.020) | 0.030 (0.020, 0.040) | 0.267 |  | 0.010 (0.010, 0.020) | 0.200 (0.200, 0.300) | <0.001 |
| RBC-Blood (10^12^/L) | 3.870 (3.262, 4.305) | 4.450 (4.230, 4.710) | 0.768 |  | 4.060 (3.562, 4.549) | 4.300 (3.761, 4.648) | 0.060 |
| HGB (g/L) | 113.000 (93.000, 127.000) | 134.000 (127.000, 141.000) | <0.001 |  | 122.000 (107.000, 136.000) | 132.000 (121.768, 142.000) | 0.745 |
| HCT (%) | 0.360 (0.290, 0.420) | 0.410 (0.390, 0.430) | <0.001 |  | 0.370 (0.326, 0.411) | 0.389 (0.361, 0.430) | 0.332 |
| MCV (fL) | 90.750 (87.000, 94.500) | 92.550 (89.800, 95.400) | 0.135 |  | 91.845 (88.525, 95.275) | 89.350 (84.200, 93.375) | <0.001 |
| MCH (pg) | 29.615 (28.200, 30.900) | 30.500 (29.400, 31.300) | <0.001 |  | 30.400 (28.900, 31.375) | 29.000 (24.098, 31.075) | <0.001 |
| MCHC (g/L) | 324.955 (316.000, 331.000) | 327.000 (322.000, 333.000) | <0.001 |  | 328.000 (319.250, 337.000) | 326.500 (316.000, 335.000) | <0.001 |
| RDW-SD (fL) | 44.185 (41.225, 48.575) | 41.800 (40.500, 43.400) | <0.001 |  | 45.200 (42.400, 49.975) | 42.250 (39.725, 44.400) | <0.001 |
| PLT (10^9^/L) | 176.000 (116.662, 228.000) | 224.591 (190.250, 259.000) | <0.001 |  | 182.075 (134.250, 230.750) | 191.000 (144.670, 241.000) | 0.734 |
| MPV (fL) | 10.200 (9.200, 11.100) | 10.400 (9.700, 11.200) | 0.248 |  | 10.900 (9.990, 12.000) | 11.500 (9.100, 12.300) | <0.001 |
| PCT-Blood (%) | 0.180 (0.136, 0.240) | 0.240 (0.210, 0.260) | 0.169 |  | 0.200 (0.150, 0.240) | 0.240 (0.220, 0.280) | <0.001 |
| PDW (%) | 16.100 (15.700, 16.500) | 16.200 (15.900, 16.400) | 0.126 |  | 15.060 (12.500, 16.300) | 14.400 (12.700, 16.175) | 0.105 |
| P-LCR (%) | 27.700 (21.800, 34.600) | 29.070 (23.700, 34.875) | 0.065 |  | 32.500 (24.400, 40.775) | 35.550 (27.803, 43.350) | 0.185 |
| ALT (U/L) | 21.700 (13.500, 33.686) | 16.226 (12.500, 22.275) | <0.001 |  | 15.000 (11.000, 23.000) | 14.000 (12.700, 16.175) | 0.006 |
| AST (U/L) | 26.100 (18.900, 40.632) | 19.500 (16.525, 23.300) | <0.001 |  | 20.000 (16.000, 26.000) | 17.370 (15.000, 23.750) | 0.008 |
| AST/ALT | 1.300 (0.980, 1.848) | 1.170 (0.950, 1.430) | <0.001 |  | 1.310 (1.000, 1.666) | 1.368 (1.000, 1.704) | <0.001 |
| TP (g/L) | 69.200 (62.925, 73.898) | 74.186 (71.500, 76.799) | <0.001 |  | 64.500 (56.900, 71.922) | 71.600 (66.815, 74.900) | <0.001 |
| ALB (g/L) | 36.744 (31.700, 40.600) | 45.400 (43.900, 47.175) | <0.001 |  | 37.950 (31.600, 41.900) | 44.873 (42.863, 46.294) | <0.001 |
| GLO (g/L) | 31.539 (26.625, 35.900) | 28.652 (26.200, 30.800) | <0.001 |  | 27.100 (22.125, 31.500) | 25.645 (24.514, 27.775) | <0.001 |
| A/G | 1.175 (0.920, 1.418) | 1.563 (1.460, 1.760) | <0.001 |  | 1.400 (1.200, 1.600) | 1.368 (1.000, 1.704) | <0.001 |
| TBIL (umol/L) | 9.150 (6.900, 11.885) | 12.665 (10.600, 15.300) | 0.002 |  | 8.400 (6.100, 11.600) | 12.296 (8.854, 15.675) | 0.327 |
| DBIL (umol/L) | 3.000 (2.000, 4.200) | 3.110 (2.300, 4.300) | 0.013 |  | 2.900 (2.100, 4.000) | 3.900 (3.100, 4.984) | 0.913 |
| IBIL (umol/L) | 6.100 (4.525, 7.900) | 9.443 (7.700, 11.662) | <0.001 |  | 5.700 (4.000, 7.875) | 8.151 (6.568, 11.075) | 0.015 |
| Urea-Blood (mmol/L) | 5.795 (4.250, 8.102) | 4.805 (4.043,5.551) | <0.001 |  | 8.076 (6.295, 10.925) | 4.886 (4.235, 5.750) | <0.001 |
| UA (umol/L) | 348.811 (272.675, 414.425) | 293.400 (251.125, 342.900) | <0.001 |  | 351.100 (304.715, 400.750) | 254.000 (215.250, 322.750) | <0.001 |
| Crea-Blood (U/L) | 62.800 (51.300, 77.325) | 55.900 (50.225, 62.825) | 0.658 |  | 13.840 (6.730, 41.325) | 51.500 (16.750, 62.900) | <0.001 |
| FT3 (pmol/L) | 2.095 (1.672, 2.470) | 2.540 (2.340, 2.770) | 0.411 |  | 4.090 (3.422, 4.670) | 4.730 (4.270, 5.200) | <0.001 |
| FT4 (pmol/L) | 1.040 (0.860, 1.210) | 1.090 (0.960, 1.197) | 0.140 |  | 1.442 (1.261, 1.692) | 1.538 (1.326, 1.712) | 0.132 |
| TSH (uIU/ml) | 2.715 (1.307, 4.682) | 2.502 (1.716, 3.705) | <0.001 |  | 2.394 (1.233, 4.410) | 2.668 (1.622, 4.216) | 0.946 |
| GGT (U/L) | 29.483 (16.800, 53.207) | 15.400 (11.520,22.675) | <0.001 |  | 30.715 (19.225, 49.330) | - | - |
| ALP-Blood (U/L) | 68.000 (53.400, 85.514) | 65.292 (54.620,76.375) | <0.001 |  | 75.235 (62.000, 95.000) | - | - |
| TgAb (uIU/ml) | 73.200 (0.110, 229.746) | 0.980 (0.060, 153.576) | <0.001 |  | - | - | - |
| TPOAb (uIU/ml) | 12.905 (0.030, 48.060) | 0.030 (0.020, 112.065) | 0.004 |  | - | - | - |
| TBA (umol/L) | 4.200 (2.500, 6.875) | - | - |  | 4.474 (3.000, 6.675) |  |  |
| K (mmol/L) | 3.970 (3.730, 4.230) | - | - |  | 3.864 (3.660, 4.099) | - | - |
| Cl (mmol/L) | 108.500 (106.300, 111.000) | - | - |  | 104.111 (40.573, 108.000) | - | - |
| Ca (mmol/L) | 2.190 (2.072, 2.301) | - | - |  | 2.360 (2.141, 64.269) | - | - |
| CO_2_-Blood (mmol/L) | 23.800 (21.500, 25.875) | - | - |  | 25.300 (22.200, 27.200) | - | - |
| IgA (g/L) | 2.770 (2.140, 3.420) | - | - |  | 2.350 (1.770, 2.873) | - | - |
| IgG (g/L) | 15.312 (12.000, 19.920) | - | - |  | 11.373 (8.518, 13.984) | - | - |
| IgM (g/L) | 1.081 (0.790, 1.510) | - | - |  | 0.994 (0.700, 1.304) | - | - |
| C3 (g/L) | 0.686 (0.395, 0.913) | - | - |  | 1.040 (0.878, 1.188) | - | - |
| C4 (g/L) | 0.617 (0.081, 0.254) | - | - |  | 0.201 (0.156, 0.260) | - | - |
| ANA (+), n (%) | 464 (73.100%) | - | - |  | - | - | - |
| Anti-dsDNA (+), n (%) | 163 (25.700%) | - | - |  | - | - | - |
| Anti-Sm (+), n (%) | 177 (27.900%) | - | - |  | - | - | - |
| Anti-SSA (+), n (%) | 328 (51.700%) | - | - |  | - | - | - |
| Anti-SSB (+), n (%) | 99 (15.600%) | - | - |  | - | - | - |
| Anti-RNP (+), n (%) | 246 (38.800%) | - | - |  | - | - | - |
| Anti-Jo01 (+), n (%) | 12 (1.800%) | - | - |  | - | - | - |
| Overlap syndrome, n (%) | 42 (6.600%) | - |  |  | - |  |  |

SLE, Systemic lupus erythematosus; HC, Healthy control; ANA, Antinuclear Antibody; Anti-dsDNA, Anti-double stranded DNA Antibody; Anti-Sm, Anti-Smith Antibody; Anti-SSA, Anti-Sjögren syndrome A Antibody; Anti-SSB, Anti-Sjögren syndrome B Antibody; Anti-RNP, Anti-ribonucleoprotein Antibody; Anti-Jo01, Anti-Jo-1 Antibody; WBC-Blood, White Blood Cells in Blood; NEU-Blood, Neutrophils in Blood; LYM, Lymphocytes; MONO, Monocytes; EOS, Eosinophils; BASO, Basophils; RBC-Blood, Red Blood Cells in Blood; HGB, Hemoglobin; HCT, Hematocrit; MCV, Mean Corpuscular Volume; MCH, Mean Corpuscular Hemoglobin; MCHC, Mean Corpuscular Hemoglobin Concentration; RDW-SD, Red Cell Distribution Width-Standard Deviation; PLT, Platelets; MPV, Mean Platelet Volume; PCT-Blood, Platelet Count in Blood; PDW, Platelet Distribution Width; P-LCR, Platelet Large Cell Ratio; ALT, Alanine Aminotransferase; AST, Aspartate Aminotransferase; AST/ALT, Aspartate Aminotransferase to Alanine Aminotransferase Ratio; TP, Total Protein; ALB, Albumin; GLO, Globulin; A/G, Albumin to Globulin Ratio; TBIL, Total Bilirubin; DBIL, Direct Bilirubin; IBIL, Indirect Bilirubin; TBA, Total Bile Acids; GGT, gamma-Glutamyltransferase; ALP-Blood, Alkaline Phosphatase in Blood; UA, Uric Acid; CO_2_-Blood, Carbon Dioxide in Blood; IgA, Immunoglobulin A; IgG, Immunoglobulin G; IgM, Immunoglobulin M; C3, Complement 3; C4, Complement 4; FT3, Free Triiodothyronine; FT4, Free Thyroxine; TSH, Thyroid Stimulating Hormone; TgAb, Thyroglobulin Antibody; TPOAb, Thyroid Peroxidase Antibody.

Supplementary table 2 Quality assessment of New Castle-Ottawa scale (NOS) scale of the included studies.

|  | Selection | | | |  | Comparability |  | Outcome | | | Score |
| --- | --- | --- | --- | --- | --- | --- | --- | --- | --- | --- | --- |
| Study | Representativeness of the exposed cohort (Patients) | Selection of the non-exposed cohort (Other means of AO assessment | Ascertainment of exposure | Demonstration that outcome of interest was not present at start of the study |  | Comparability of cohorts based on the design or analysis controlled for confounders |  | Assessment of outcome | Was follow-up time long enough for outcomes to occur | Adequacy of follow-up of cohorts |  |
| Al-Awadhi, et al. 2008 | 1 | 1 | 1 | 1 |  | 0 |  | 1 | 1 | 0 | 6 |
| Domingues, et al. 2017 | 1 | 1 | 0 | 1 |  | 2 |  | 1 | 1 | 0 | 7 |
| Watad, et al. 2016 | 1 | 1 | 1 | 1 |  | 2 |  | 1 | 1 | 0 | 8 |
| Watad, et al. 2016 | 1 | 1 | 1 | 1 |  | 1 |  | 1 | 1 | 0 | 7 |
| Appenzeller, et al. 2009 | 1 | 1 | 0 | 1 |  | 1 |  | 1 | 0 | 0 | 5 |
| Antonelli, et al. 2009 | 1 | 1 | 1 | 1 |  | 1 |  | 1 | 1 | 0 | 7 |
| Liu, et al. 2019 | 1 | 1 | 1 | 1 |  | 1 |  | 1 | 1 | 0 | 7 |
| Lin, et al. 2015 | 1 | 1 | 1 | 0 |  | 1 |  | 1 | 1 | 0 | 6 |
| Kumar, et al. 2012 | 1 | 1 | 1 | 1 |  | 1 |  | 1 | 1 | 0 | 7 |
| Mader, et al. 2007 | 1 | 0 | 0 | 1 |  | 0 |  | 1 | 1 | 0 | 4 |
| Li, et al. 2021 | 1 | 1 | 1 | 1 |  | 1 |  | 1 | 1 | 0 | 7 |
| Posselt, et al. 2017 | 1 | 1 | 0 | 1 |  | 2 |  | 1 | 1 | 0 | 7 |
| Zhang, et al. 2023 | 1 | 1 | 1 | 1 |  | 1 |  | 1 | 1 | 0 | 7 |
| Ni, et al. 2022 | 1 | 1 | 1 | 1 |  | 1 |  | 1 | 1 | 0 | 7 |
| Athanassiou, et al. 2023 | 1 | 0 | 1 | 1 |  | 0 |  | 1 | 1 | 0 | 5 |
| Luzhou cohort | 1 | 1 | 1 | 1 |  | 1 |  | 1 | 1 | 0 | 7 |
| Enshi cohort | 1 | 1 | 1 | 1 |  | 1 |  | 1 | 1 | 0 | 7 |
| Parente Costa, et al. 2009 | 1 | 1 | 1 | 1 |  | 0 |  | 1 | 1 | 0 | 6 |

Supplementary table 3 Univariate logistic regression analysis of variables in SLE patients with clinical hyperthyroidism in Luzhou cohort.

| Variables | OR (95% CI) | P value |
| --- | --- | --- |
| Sex | 2.431 (0.531, 8.357) | 0.190 |
| Age (year) | 1.002 (0.964, 1.041) | 0.926 |
| LYM (10^9^/L) | 2.154 (1.131, 3.831) | 0.010 |
| MCV (fL) | 0.863 (0.814, 0.911) | <0.001 |
| MCH (pg) | 0.765 (0.661, 0.870) | <0.001 |
| RDW-SD (fL) | 0.906 (0.859, 0.960) | <0.001 |
| AST/ALT | 0.099 (0.018, 0.406) | 0.004 |
| ALP-Blood (U/L) | 1.006 (1.001, 1.010) | 0.009 |
| TPOAb (uIU/ml) | 1.004 (1.002, 1.005) | <0.001 |
| Overlap syndrome (+) | 0.000 (NA, 8.266^47^) | 0.992 |
| ANA (+) | 0.358 (0.111, 1.159) | 0.079 |
| Anti-dsDNA (+) | 0.573 (0.087, 2.201) | 0.475 |
| Anti-Sm (+) | 0.858 (0.189, 2.915) | 0.820 |
| Anti-SSA (+) | 0.305 (0.067, 1.032) | 0.077 |
| Anti-SSB (+) | 0.000 (NA, 5.510^28^) | 0.988 |
| Anti-RNP (+) | 0.785 (0.208, 2.521) | 0.695 |
| Anti-Jo01 (+) | 0.000 (NA, 1.344^23^) | 0.990 |
| WBC-Blood (10^9^/L) | 1.028 (0.919, 1.084) | 0.415 |
| NEU-Blood (10^9^/L) | 0.999 (NA, 1.022) | 0.968 |
| MONO (10^9^/L) | 1.010 (NA, 1.080) | 0.884 |
| EOS (10^9^/L) | 0.292 (0.000, 0.575) | 0.769 |
| BASO (10^9^/L) | 0.000 (0.000, 1.108) | 0.540 |
| RBC-Blood (10^12^/L) | 1.000 (NA, 1.010) | 0.994 |
| HGB (g/L) | 1.002 (0.988, 1.008) | 0.542 |
| HCT (%) | 0.998 (0.919, 1.040) | 0.938 |
| MCHC (g/L) | 1.002 (0.983, 1.035) | 0.901 |
| PLT (10^9^/L) | 1.002 (0.995, 1.008) | 0.641 |
| MPV (fL) | 0.996 (0.756, 1.081) | 0.966 |
| PCT-Blood (%) | 0.587 (0.002, 0.914) | 0.763 |
| PDW (%) | 0.884 (0.722, 1.042) | 0.298 |
| P-LCR (%) | 1.026 (0.973, 1.077) | 0.309 |
| ALT (U/L) | 1.000 (0.986, 1.003) | 0.956 |
| AST (U/L) | 0.992 (0.960, 1.002) | 0.503 |
| TP (g/L) | 1.006 (0.954, 1.038) | 0.775 |
| ALB (g/L) | 1.016 (0.937, 1.113) | 0.714 |
| GLO (g/L) | 1.002 (0.926, 1.071) | 0.962 |
| A/G | 0.855 (0.166, 2.490) | 0.839 |
| TBIL (umol/L) | 1.005 (0.950, 1.026) | 0.758 |
| DBIL (umol/L) | 1.007 (0.916, 1.037) | 0.759 |
| IBIL (umol/L) | 1.008 (0.896, 1.061) | 0.827 |
| TBA (umol/L) | 1.003 (0.966, 1.015) | 0.791 |
| GGT (U/L) | 0.995 (0.977, 1.003) | 0.518 |
| Urea-Blood (mmol/L) | 0.974 (0.819, 1.015) | 0.667 |
| UA (umol/L) | 0.999 (0.994, 1.003) | 0.656 |
| Crea-Blood (U/L) | 0.980 (0.947, 1.001) | 0.192 |
| K (mmol/L) | 0.961 (0.296, 1.067) | 0.928 |
| Cl (mmol/L) | 0.990 (0.946, 1.112) | 0.780 |
| Ca (mmol/L) | 1.061 (NA, 1.303) | 0.722 |
| CO_2_-Blood (mmol/L) | 1.005 (0.872, 1.187) | 0.948 |
| IgA (g/L) | 1.342 (0.959, 1.763) | 0.052 |
| IgG (g/L) | 0.963 (0.866, 1.047) | 0.435 |
| IgM (g/L) | 0.937 (0.368, 1.749) | 0.870 |
| C3 (g/L) | 1.384 (0.256, 7.157) | 0.700 |
| C4 (g/L) | 4.452 (0.708, 17.902) | 0.059 |
| FT3 (pmol/L) | 1.001 (NA, 1.006) | 0.712 |
| FT4 (pmol/L) | 1.005 (NA, 1.019) | 0.589 |
| TSH (uIU/ml) | 0.000 (0.000, 0.001) | 0.002 |
| TgAb (uIU/ml) | 1.000 (0.999, 1.001) | 0.733 |

LYM, Lymphocytes; MCV, Mean Corpuscular Volume; MCH, Mean Corpuscular Hemoglobin; RDW-SD, Red Cell Distribution Width-Standard Deviation; AST/ALT, Aspartate Aminotransferase to Alanine Aminotransferase Ratio; ALP-Blood, Alkaline Phosphatase in Blood; TPOAb, Thyroid Peroxidase Antibody; ANA, Antinuclear Antibody; Anti-dsDNA, Anti-double stranded DNA Antibody; Anti-Sm, Anti-Smith Antibody; Anti-SSA, Anti-Sjögren syndrome A Antibody; Anti-SSB, Anti-Sjögren syndrome B Antibody; Anti-RNP, Anti-ribonucleoprotein Antibody; Anti-Jo01, Anti-Jo-1 Antibody; WBC-Blood, White Blood Cells in Blood; NEU-Blood, Neutrophils in Blood; MONO, Monocytes; EOS, Eosinophils; BASO, Basophils; RBC-Blood, Red Blood Cells in Blood; HGB, Hemoglobin; HCT, Hematocrit; MCHC, Mean Corpuscular Hemoglobin Concentration; PLT, Platelets; MPV, Mean Platelet Volume; PCT-Blood, Platelet Count in Blood; PDW, Platelet Distribution Width; P-LCR, Platelet Large Cell Ratio; ALT, Alanine Aminotransferase; AST, Aspartate Aminotransferase; TP, Total Protein; ALB, Albumin; GLO, Globulin; A/G, Albumin to Globulin Ratio; TBIL, Total Bilirubin; DBIL, Direct Bilirubin; IBIL, Indirect Bilirubin; TBA, Total Bile Acids; GGT, gamma-Glutamyltransferase; UA, Uric Acid; CO_2_-Blood, Carbon Dioxide in Blood; IgA, Immunoglobulin A; IgG, Immunoglobulin G; IgM, Immunoglobulin M; C3, Complement 3; C4, Complement 4; FT3, Free Triiodothyronine; FT4, Free Thyroxine; TSH, Thyroid Stimulating Hormone; TgAb, Thyroglobulin Antibody.

Supplementary table 4 Multivariate logistic regression analysis of variables in SLE patients with clinical hyperthyroidism in Luzhou cohort.

| Variables | Coefficient β | OR (95% CI) | *Z* | P value |
| --- | --- | --- | --- | --- |
| MCV (fL) | -0.143 | 0.867 (0.805, 0.925) | -4.113 | <0.001 |
| RDW-SD (fL) | -0.076 | 0.927 (0.850, 1.030) | -1.597 | 0.110 |
| AST/ALT | -2.256 | 0.105 (0.015, 0.443) | -2.679 | 0.007 |
| TPOAb (uIU/ml) | 0.004 | 1.004 (1.002, 1.006) | 3.577 | <0.001 |

MCV, Mean Corpuscular Volume; RDW-SD, Red Cell Distribution Width-Standard Deviation; AST/ALT, Aspartate Aminotransferase to Alanine Aminotransferase Ratio; TPOAb, Thyroid Peroxidase Antibody.

Supplementary table 5 Univariate logistic regression analysis of variables in SLE patients with subclinical hyperthyroidism in Luzhou cohort.

| Variables | OR (95% CI) | P value |
| --- | --- | --- |
| Sex | 2.093 (0.581, 6.015) | 0.203 |
| Age (year) | 1.017 (0.986, 1.050) | 0.297 |
| Overlap syndrome (+) | 0.825 (0.045, 4.175) | 0.853 |
| ANA (+) | 0.565 (0.219, 1.560) | 0.247 |
| Anti-dsDNA (+) | 0.165 (0.009, 0.812) | 0.081 |
| Anti-Sm (+) | 0.732 (0.205, 2.072) | 0.586 |
| Anti-SSA (+) | 1.171 (0.456, 3.106) | 0.742 |
| Anti-SSB (+) | 1.083 (0.248, 3.360) | 0.901 |
| Anti-RNP (+) | 0.598 (0.190, 1.609) | 0.335 |
| Anti-Jo01 (+) | 0.000 (NA, 1.227^29^) | 0.990 |
| WBC-Blood (10^9^/L) | 1.009 (0.891, 1.069) | 0.834 |
| NEU-Blood (10^9^/L) | 0.998 (NA, 1.020) | 0.920 |
| LYM (10^9^/L) | 0.687 (0.272, 1.481) | 0.388 |
| MONO (10^9^/L) | 0.995 (NA, 1.076) | 0.954 |
| EOS (10^9^/L) | 0.002 (0.000, 0.558) | 0.236 |
| BASO (10^9^/L) | 0.838 (NA, 1.159) | 0.906 |
| RBC-Blood (10^12^/L) | 0.995 (NA, 1.009) | 0.905 |
| HGB (g/L) | 1.000 (0.983, 1.007) | 0.993 |
| HCT (%) | 1.004 (0.951, 1.038) | 0.845 |
| MCV (fL) | 1.003 (0.959, 1.023) | 0.815 |
| MCH (pg) | 1.071 (0.951, 1.175) | 0.184 |
| MCHC (g/L) | 1.017 (0.991, 1.042) | 0.191 |
| RDW-SD (fL) | 1.027 (0.992, 1.057) | 0.065 |
| PLT (10^9^/L) | 0.999 (0.993, 1.004) | 0.695 |
| MPV (fL) | 1.025 (0.894, 1.086) | 0.487 |
| PCT-Blood (%) | 0.265 (0.001, 1.075) | 0.562 |
| PDW (%) | 0.989 (0.828, 1.050) | 0.855 |
| P-LCR (%) | 1.041 (0.998, 1.082) | 0.050 |
| ALT (U/L) | 1.003 (1.000, 1.005) | 0.020 |
| AST (U/L) | 1.002 (1.000, 1.004) | 0.015 |
| AST/ALT | 1.018 (0.850, 1.076) | 0.672 |
| TP (g/L) | 1.004 (0.960, 1.033) | 0.850 |
| ALB (g/L) | 1.001 (0.938, 1.076) | 0.970 |
| GLO (g/L) | 1.014 (0.953, 1.070) | 0.651 |
| A/G | 0.774 (0.201, 2.113) | 0.695 |
| TBIL (umol/L) | 1.009 (0.979, 1.026) | 0.353 |
| DBIL (umol/L) | 1.006 (0.932, 1.033) | 0.786 |
| IBIL (umol/L) | 1.032 (0.979, 1.069) | 0.120 |
| TBA (umol/L) | 1.000 (0.962, 1.013) | 0.986 |
| GGT (U/L) | 1.002 (0.999, 1.005) | 0.075 |
| ALP-Blood (U/L) | 1.001 (0.993, 1.006) | 0.681 |
| Urea-Blood (mmol/L) | 0.983 (0.872, 1.013) | 0.691 |
| UA (umol/L) | 0.999 (0.995, 1.002) | 0.603 |
| Crea-Blood (U/L) | 1.001 (0.995, 1.004) | 0.582 |
| K (mmol/L) | 0.630 (0.219, 1.005) | 0.361 |
| Cl (mmol/L) | 0.972 (0.934, 1.022) | 0.120 |
| Ca (mmol/L) | 0.427 (0.031, 1.217) | 0.529 |
| CO_2_-Blood (mmol/L) | 1.029 (0.909, 1.182) | 0.668 |
| IgA (g/L) | 1.086 (0.762, 1.439) | 0.608 |
| IgG (g/L) | 1.032 (0.968, 1.087) | 0.283 |
| IgM (g/L) | 1.162 (0.625, 1.823) | 0.575 |
| C3 (g/L) | 0.421 (0.096, 1.694) | 0.234 |
| C4 (g/L) | 0.104 (0.001, 1.850) | 0.236 |
| FT3 (pmol/L) | 0.986 (NA, 1.007) | 0.896 |
| FT4 (pmol/L) | 0.994 (NA, 1.016) | 0.867 |
| TSH (uIU/ml) | 0.004 (0.000, 0.025) | <0.001 |
| TgAb (uIU/ml) | 1.000 (0.999, 1.001) | 0.584 |
| TPOAb (uIU/ml) | 0.999 (0.993, 1.002) | 0.759 |

SLE, Systemic lupus erythematosus; ANA, Antinuclear Antibody; Anti-dsDNA, Anti-double stranded DNA Antibody; Anti-Sm, Anti-Smith Antibody; Anti-SSA, Anti-Sjögren syndrome A Antibody; Anti-SSB, Anti-Sjögren syndrome B Antibody; Anti-RNP, Anti-ribonucleoprotein Antibody; Anti-Jo01, Anti-Jo-1 Antibody; WBC-Blood, White Blood Cells in Blood; NEU-Blood, Neutrophils in Blood; LYM, Lymphocytes; MONO, Monocytes; EOS, Eosinophils; BASO, Basophils; RBC-Blood, Red Blood Cells in Blood; HGB, Hemoglobin; HCT, Hematocrit; MCV, Mean Corpuscular Volume; MCH, Mean Corpuscular Hemoglobin; MCHC, Mean Corpuscular Hemoglobin Concentration; RDW-SD, Red Cell Distribution Width-Standard Deviation; PLT, Platelets; MPV, Mean Platelet Volume; PCT-Blood, Platelet Count in Blood; PDW, Platelet Distribution Width; P-LCR, Platelet Large Cell Ratio; ALT, Alanine Aminotransferase; AST, Aspartate Aminotransferase; AST/ALT, Aspartate Aminotransferase to Alanine Aminotransferase Ratio; TP, Total Protein; ALB, Albumin; GLO, Globulin; A/G, Albumin to Globulin Ratio; TBIL, Total Bilirubin; DBIL, Direct Bilirubin; IBIL, Indirect Bilirubin; TBA, Total Bile Acids; GGT, gamma-Glutamyltransferase; ALP-Blood, Alkaline Phosphatase in Blood; UA, Uric Acid; CO_2_-Blood, Carbon Dioxide in Blood; IgA, Immunoglobulin A; IgG, Immunoglobulin G; IgM, Immunoglobulin M; C3, Complement 3; C4, Complement 4; FT3, Free Triiodothyronine; FT4, Free Thyroxine; TSH, Thyroid Stimulating Hormone; TgAb, Thyroglobulin Antibody; TPOAb, Thyroid Peroxidase Antibody.

Supplementary table 6 Univariate logistic regression analysis of variables in SLE patients with clinical hypothyroidism in Luzhou cohort.

| Variables | OR (95% CI) | P value |
| --- | --- | --- |
| Sex | 0.477 (0.114, 1.352) | 0.225 |
| Age (year) | 0.995 (0.975, 1.014) | 0.587 |
| Anti-RNP (+) | 0.472 (0.225, 0.919) | 0.035 |
| ALB (g/L) | 0.951 (0.913, 0.991) | 0.016 |
| Cl (mmol/L) | 1.112 (1.030, 1.203) | 0.007 |
| Ca (mmol/L) | 0.136 (0.025, 0.747) | 0.021 |
| FT3 (pmol/L) | 0.257 (0.149, 0.435) | <0.001 |
| TSH (uIU/ml) | 1.056 (1.038, 1.081) | <0.001 |
| TPOAb (uIU/ml) | 1.002 (1.001, 1.004) | <0.001 |
| Overlap syndrome (+) | 1.378 (0.399, 3.641) | 0.559 |
| ANA (+) | 0.665 (0.357, 1.281) | 0.208 |
| Anti-dsDNA (+) | 0.790 (0.364, 1.572) | 0.523 |
| Anti-Sm (+) | 0.700 (0.323, 1.392) | 0.334 |
| Anti-SSA (+) | 0.699 (0.378, 1.276) | 0.247 |
| Anti-SSB (+) | 0.494 (0.146, 1.258) | 0.188 |
| Anti-Jo01 (+) | 1.166 (0.063, 6.198) | 0.885 |
| WBC-Blood (10^9^/L) | 1.006 (0.932, 1.055) | 0.843 |
| NEU-Blood (10^9^/L) | 1.014 (0.999, 1.030) | 0.051 |
| LYM (10^9^/L) | 1.298 (0.827, 1.946) | 0.227 |
| MONO (10^9^/L) | 0.978 (NA, 1.063) | 0.838 |
| EOS (10^9^/L) | 0.891 (NA, 1.049) | 0.864 |
| BASO (10^9^/L) | 0.816 (NA, 1.087) | 0.859 |
| RBC-Blood (10^12^/L) | 0.842 (0.615, 1.007) | 0.284 |
| HGB (g/L) | 0.997 (0.986, 1.005) | 0.645 |
| HCT (%) | 1.010 (0.982, 1.033) | 0.425 |
| MCV (fL) | 1.009 (0.990, 1.026) | 0.227 |
| MCH (pg) | 1.072 (0.993, 1.155) | 0.061 |
| MCHC (g/L) | 1.006 (0.990, 1.025) | 0.577 |
| RDW-SD (fL) | 1.010 (0.978, 1.035) | 0.493 |
| PLT (10^9^/L) | 1.000 (0.996, 1.003) | 0.934 |
| MPV (fL) | 1.001 (0.874, 1.063) | 0.978 |
| PCT-Blood (%) | 1.046 (0.925, 1.140) | 0.300 |
| PDW (%) | 1.019 (0.971, 1.053) | 0.303 |
| P-LCR (%) | 1.015 (0.986, 1.043) | 0.305 |
| ALT (U/L) | 1.000 (0.997, 1.003) | 0.722 |
| AST (U/L) | 1.001 (0.999, 1.003) | 0.167 |
| AST/ALT | 0.984 (0.767, 1.052) | 0.794 |
| TP (g/L) | 0.995 (0.966, 1.020) | 0.736 |
| GLO (g/L) | 0.977 (0.936, 1.017) | 0.272 |
| A/G | 0.675 (0.284, 1.450) | 0.360 |
| TBIL (umol/L) | 0.984 (0.934, 1.011) | 0.444 |
| DBIL (umol/L) | 0.965 (0.858, 1.016) | 0.440 |
| IBIL (umol/L) | 0.992 (0.928, 1.034) | 0.774 |
| TBA (umol/L) | 0.996 (0.967, 1.008) | 0.651 |
| GGT (U/L) | 1.002 (0.999, 1.004) | 0.105 |
| ALP-Blood (U/L) | 1.001 (0.996, 1.005) | 0.548 |
| Urea-Blood (mmol/L) | 1.001 (0.977, 1.010) | 0.928 |
| UA (umol/L) | 1.002 (1.000, 1.004) | 0.040 |
| Crea-Blood (U/L) | 1.001 (0.998, 1.004) | 0.304 |
| K (mmol/L) | 0.998 (NA, 1.036) | 0.958 |
| CO_2_-Blood (mmol/L) | 0.995 (0.920, 1.082) | 0.898 |
| IgA (g/L) | 0.813 (0.614, 1.041) | 0.126 |
| IgG (g/L) | 0.968 (0.919, 1.013) | 0.190 |
| IgM (g/L) | 1.164 (0.798, 1.599) | 0.385 |
| C3 (g/L) | 0.811 (0.331, 1.945) | 0.641 |
| C4 (g/L) | 0.636 (0.110, 2.363) | 0.556 |
| FT4 (pmol/L) | 0.000 (0.000, 0.000) | <0.001 |
| TgAb (uIU/ml) | 1.001 (1.000, 1.001) | 0.003 |

Anti-RNP, Anti-ribonucleoprotein antibody; ALB, Albumin; FT3, Free Triiodothyronine; TSH, Thyroid Stimulating Hormone; TPOAb, Thyroid Peroxidase Antibody; ANA, Antinuclear Antibody; Anti-dsDNA, Anti-double stranded DNA Antibody; Anti-Sm, Anti-Smith Antibody; Anti-SSA, Anti-Sjögren syndrome A Antibody; Anti-SSB, Anti-Sjögren syndrome B Antibody; Anti-Jo01, Anti-Jo-1 Antibody; WBC-Blood, White Blood Cells in Blood; NEU-Blood, Neutrophils in Blood; LYM, Lymphocytes; MONO, Monocytes; EOS, Eosinophils; BASO, Basophils; RBC-Blood, Red Blood Cells in Blood; HGB, Hemoglobin; HCT, Hematocrit; MCV, Mean Corpuscular Volume; MCH, Mean Corpuscular Hemoglobin; MCHC, Mean Corpuscular Hemoglobin Concentration; RDW-SD, Red Cell Distribution Width-Standard Deviation; PLT, Platelets; MPV, Mean Platelet Volume; PCT-Blood, Platelet Count in Blood; PDW, Platelet Distribution Width; P-LCR, Platelet Large Cell Ratio; ALT, Alanine Aminotransferase; AST, Aspartate Aminotransferase; AST/ALT, Aspartate Aminotransferase to Alanine Aminotransferase Ratio; TP, Total Protein; GLO, Globulin; A/G, Albumin to Globulin Ratio; TBIL, Total Bilirubin; DBIL, Direct Bilirubin; IBIL, Indirect Bilirubin; TBA, Total Bile Acids; GGT, gamma-Glutamyltransferase; ALP-Blood, Alkaline Phosphatase in Blood; UA, Uric Acid; CO_2_-Blood, Carbon Dioxide in Blood; IgA, Immunoglobulin A; IgG, Immunoglobulin G; IgM, Immunoglobulin M; C3, Complement 3; C4, Complement 4; FT4, Free Thyroxine; TgAb, Thyroglobulin Antibody.

Supplementary table 7 Multivariate logistic regression analysis of variables in SLE patients with clinical hypothyroidism in Luzhou cohort.

| Variables | Coefficient β | OR (95% CI) | Z | P value |
| --- | --- | --- | --- | --- |
| Cl (mmol/L) | 0.160 | 1.173 (1.057, 1.307) | 2.957 | 0.003 |
| FT3 (pmol/L) | -1.354 | 0.258 (0.114, 0.560) | -3.353 | 0.001 |
| TSH (uIU/ml) | 0.232 | 1.261 (1.182, 1.357) | 6.615 | <0.001 |

FT3, Free Triiodothyronine; TSH, Thyroid Stimulating Hormone.

Supplementary table 8 Univariate logistic regression analysis of variables in SLE patients with subclinical hypothyroidism in Luzhou cohort.

| Variables | OR (95% CI) | P value |
| --- | --- | --- |
| Sex | 0.733 (0.315, 1.501) | 0.430 |
| Age (year) | 1.012 (0.997, 1.028) | 0.119 |
| Anti-Jo01 (+) | 3.437 (0.900, 11.179) | 0.048 |
| TP (g/L) | 0.975 (0.952, 0.997) | 0.030 |
| ALB (g/L) | 0.958 (0.928, 0.989) | 0.008 |
| TBIL (umol/L) | 0.935 (0.880, 0.982) | 0.018 |
| DBIL (umol/L) | 0.889 (0.781, 0.978) | 0.046 |
| IBIL (umol/L) | 0.904 (0.828, 0.972) | 0.015 |
| Cl (mmol/L) | 1.071 (1.012, 1.137) | 0.022 |
| Ca (mmol/L) | 0.144 (0.038, 0.535) | 0.004 |
| Overlap syndrome (+) | 1.358 (0.537, 2.995) | 0.479 |
| ANA (+) | 1.018 (0.612, 1.749) | 0.946 |
| Anti-dsDNA (+) | 1.125 (0.660, 1.865) | 0.655 |
| Anti-Sm (+) | 1.058 (0.626, 1.742) | 0.828 |
| Anti-SSA (+) | 0.898 (0.565, 1.426) | 0.648 |
| Anti-SSB (+) | 1.112 (0.578, 2.012) | 0.736 |
| Anti-RNP (+) | 1.110 (0.689, 1.769) | 0.665 |
| WBC-Blood (10^9^/L) | 0.942 (0.872, 1.007) | 0.108 |
| NEU-Blood (10^9^/L) | 0.923 (0.841, 0.996) | 0.077 |
| LYM (10^9^/L) | 0.898 (0.605, 1.291) | 0.580 |
| MONO (10^9^/L) | 0.532 (0.177, 1.012) | 0.242 |
| EOS (10^9^/L) | 0.949 (NA, 1.050) | 0.727 |
| BASO (10^9^/L) | 0.000 (0.000, 0.377) | 0.234 |
| RBC-Blood (10^12^/L) | 0.828 (0.647, 1.004) | 0.128 |
| HGB (g/L) | 1.002 (0.996, 1.007) | 0.402 |
| HCT (%) | 1.007 (0.985, 1.026) | 0.476 |
| MCV (fL) | 0.998 (0.974, 1.014) | 0.855 |
| MCH (pg) | 0.973 (0.909, 1.040) | 0.424 |
| MCHC (g/L) | 0.993 (0.982, 1.004) | 0.180 |
| RDW-SD (fL) | 0.987 (0.959, 1.012) | 0.354 |
| PLT (10^9^/L) | 1.000 (0.997, 1.002) | 0.750 |
| MPV (fL) | 0.974 (0.859, 1.044) | 0.637 |
| PCT-Blood (%) | 0.952 (0.585, 1.073) | 0.663 |
| PDW (%) | 0.940 (0.845, 1.013) | 0.240 |
| P-LCR (%) | 0.994 (0.971, 1.017) | 0.615 |
| ALT (U/L) | 0.993 (0.982, 0.999) | 0.113 |
| AST (U/L) | 0.995 (0.988, 1.000) | 0.143 |
| AST/ALT | 0.977 (0.803, 1.041) | 0.658 |
| GLO (g/L) | 0.995 (0.965, 1.025) | 0.770 |
| A/G | 0.568 (0.290, 1.071) | 0.093 |
| TBA (umol/L) | 0.982 (0.942, 1.002) | 0.254 |
| GGT (U/L) | 0.999 (0.995, 1.001) | 0.446 |
| ALP-Blood (U/L) | 0.998 (0.992, 1.002) | 0.392 |
| Urea-Blood (mmol/L) | 0.998 (0.973, 1.007) | 0.756 |
| UA (umol/L) | 1.002 (1.000, 1.003) | 0.032 |
| Crea-Blood (U/L) | 1.001 (0.999, 1.003) | 0.310 |
| K (mmol/L) | 0.990 (NA, 1.030) | 0.796 |
| CO_2_-Blood (mmol/L) | 1.030 (0.967, 1.101) | 0.370 |
| IgA (g/L) | 0.970 (0.807, 1.146) | 0.733 |
| IgG (g/L) | 0.996 (0.961, 1.028) | 0.794 |
| IgM (g/L) | 1.114 (0.829, 1.451) | 0.447 |
| C3 (g/L) | 0.839 (0.424, 1.646) | 0.612 |
| C4 (g/L) | 0.708 (0.206, 1.950) | 0.542 |
| FT3 (pmol/L) | 0.956 (0.734, 1.011) | 0.622 |
| FT4 (pmol/L) | 0.916 (0.539, 0.987) | 0.682 |
| TSH (uIU/ml) | 1.008 (0.999, 1.019) | 0.102 |
| TgAb (uIU/ml) | 1.001 (1.000, 1.001) | 0.013 |
| TPOAb (uIU/ml) | 1.001 (0.999, 1.002) | 0.292 |

Anti-Jo01, Anti-Jo-1 antibody; TP, Total Protein; ALB, Albumin; TBIL, Total Bilirubin; DBIL, Direct Bilirubin; IBIL, Indirect Bilirubin; ANA, Antinuclear Antibody; Anti-dsDNA, Anti-double stranded DNA Antibody; Anti-Sm, Anti-Smith Antibody; Anti-SSA, Anti-Sjögren syndrome A Antibody; Anti-SSB, Anti-Sjögren syndrome B Antibody; Anti-RNP, Anti-ribonucleoprotein Antibody; WBC-Blood, White Blood Cells in Blood; NEU-Blood, Neutrophils in Blood; LYM, Lymphocytes; MONO, Monocytes; EOS, Eosinophils; BASO, Basophils; RBC-Blood, Red Blood Cells in Blood; HGB, Hemoglobin; HCT, Hematocrit; MCV, Mean Corpuscular Volume; MCH, Mean Corpuscular Hemoglobin; MCHC, Mean Corpuscular Hemoglobin Concentration; RDW-SD, Red Cell Distribution Width-Standard Deviation; PLT, Platelets; MPV, Mean Platelet Volume; PCT-Blood, Platelet Count in Blood; PDW, Platelet Distribution Width; P-LCR, Platelet Large Cell Ratio; ALT, Alanine Aminotransferase; AST, Aspartate Aminotransferase; AST/ALT, Aspartate Aminotransferase to Alanine Aminotransferase Ratio; GLO, Globulin; A/G, Albumin to Globulin Ratio; TBA, Total Bile Acids; GGT, gamma-Glutamyltransferase; ALP-Blood, Alkaline Phosphatase in Blood; UA, Uric Acid; CO_2_-Blood, Carbon Dioxide in Blood; IgA, Immunoglobulin A; IgG, Immunoglobulin G; IgM, Immunoglobulin M; C3, Complement 3; C4, Complement 4; FT3, Free Triiodothyronine; FT4, Free Thyroxine; TSH, Thyroid Stimulating Hormone; TgAb, Thyroglobulin Antibody; TPOAb, Thyroid Peroxidase Antibody.

Supplementary table 9 Multivariate logistic regression analysis of variables in SLE patients with subclinical hypothyroidism in Luzhou cohort.

| Variables | Coefficient β | OR (95% CI) | Z | P value |
| --- | --- | --- | --- | --- |
| Age (year) | 0.018 | 1.018 (1.002, 1.034) | 2.167 | 0.030 |
| Anti-Jo01 (+) | 1.180 | 3.255 (0.806, 11.312) | 1.797 | 0.072 |
| DBIL (umol/L) | -0.096 | 0.909 (0.803, 0.989) | -1.765 | 0.078 |
| Cl (mmol/L) | 0.050 | 1.051 (0.991, 1.116) | 1.635 | 0.102 |
| Ca (mmol/L) | -1.910 | 0.148 (0.037, 0.581) | -2.728 | 0.006 |

Anti-Jo01, Anti-Jo-1 antibody; DBIL, Direct Bilirubin.

Supplementary table 10 Univariate logistic regression analysis of variables in SLE patients with ESS in Luzhou cohort.

| Variables | OR (95% CI) | P value |
| --- | --- | --- |
| Sex | 0.478 (0.076, 1.631) | 0.320 |
| Age (year) | 0.972 (0.947, 0.996) | 0.022 |
| Anti-dsDNA (+) | 2.509 (1.191, 5.209) | 0.014 |
| LYM (10^9^/L) | 0.226 (0.090, 0.510) | 0.001 |
| RBC-Blood (10^12^/L) | 0.552 (0.386, 0.795) | 0.001 |
| HGB (g/L) | 0.978 (0.964, 0.992) | 0.002 |
| ALB (g/L) | 0.922 (0.879, 0.967) | 0.001 |
| A/G | 0.287 (0.095, 0.825) | 0.024 |
| UA (umol/L) | 1.004 (1.002, 1.006) | <0.001 |
| Crea-Blood (U/L) | 1.003 (1.001, 1.006) | 0.009 |
| Ca (mmol/L) | 0.049 (0.006, 0.373) | 0.004 |
| CO_2_-Blood (mmol/L) | 0.855 (0.787, 0.928) | <0.001 |
| C3 (g/L) | 0.071 (0.018, 0.244) | <0.001 |
| FT3 (pmol/L) | 0.086 (0.039, 0.177) | <0.001 |
| FT4 (pmol/L) | 0.007 (0.001, 0.028) | <0.001 |
| TSH (uIU/ml) | 0.848 (0.702, 0.971) | 0.049 |
| Overlap syndrome (+) | 0.000 (0.000, 4.437^15^) | 0.988 |
| ANA (+) | 1.959 (0.803, 5.866) | 0.176 |
| Anti-Sm (+) | 0.743 (0.291, 1.671) | 0.498 |
| Anti-SSA (+) | 1.741 (0.835, 3.823) | 0.149 |
| Anti-SSB (+) | 0.792 (0.230, 2.083) | 0.670 |
| Anti-RNP (+) | 0.741 (0.329, 1.563) | 0.445 |
| Anti-Jo01 (+) | 0.000 (NA, 9.903^28^) | 0.990 |
| WBC-Blood (10^9^/L) | 0.997 (0.900, 1.056) | 0.950 |
| NEU-Blood (10^9^/L) | 0.999 (NA, 1.018) | 0.948 |
| MONO (10^9^/L) | 0.338 (0.052, 0.954) | 0.225 |
| EOS (10^9^/L) | 0.000 (0.000, 0.105) | 0.020 |
| BASO (10^9^/L) | 0.835 (NA, 1.110) | 0.876 |
| HCT (%) | 0.973 (0.905, 1.015) | 0.327 |
| MCV (fL) | 1.001 (0.964, 1.020) | 0.966 |
| MCH (pg) | 1.031 (0.931, 1.125) | 0.525 |
| MCHC (g/L) | 1.013 (0.992, 1.035) | 0.248 |
| RDW-SD (fL) | 1.006 (0.966, 1.035) | 0.756 |
| PLT (10^9^/L) | 0.996 (0.991, 1.000) | 0.067 |
| MPV (fL) | 0.848 (0.712, 1.019) | 0.076 |
| PCT-Blood (%) | 1.040 (0.873, 1.135) | 0.439 |
| PDW (%) | 1.031 (0.987, 1.065) | 0.085 |
| P-LCR (%) | 0.981 (0.944, 1.017) | 0.315 |
| ALT (U/L) | 1.001 (0.996, 1.003) | 0.603 |
| AST (U/L) | 1.001 (0.998, 1.003) | 0.267 |
| AST/ALT | 1.028 (0.947, 1.078) | 0.303 |
| TP (g/L) | 0.967 (0.934, 1.002) | 0.062 |
| GLO (g/L) | 1.015 (0.969, 1.059) | 0.506 |
| TBIL (umol/L) | 0.992 (0.939, 1.016) | 0.682 |
| DBIL (umol/L) | 0.984 (0.876, 1.023) | 0.669 |
| IBIL (umol/L) | 0.993 (0.913, 1.040) | 0.830 |
| TBA (umol/L) | 0.975 (0.893, 1.006) | 0.431 |
| GGT (U/L) | 1.000 (0.996, 1.003) | 0.781 |
| ALP-Blood (U/L) | 1.000 (0.991, 1.005) | 0.881 |
| Urea-Blood (mmol/L) | 1.004 (0.987, 1.013) | 0.442 |
| K (mmol/L) | 1.000 (NA, 1.039) | 0.994 |
| Cl (mmol/L) | 1.003 (0.960, 1.089) | 0.927 |
| IgA (g/L) | 1.001 (0.748, 1.277) | 0.997 |
| IgG (g/L) | 0.996 (0.941, 1.046) | 0.884 |
| IgM (g/L) | 1.268 (0.828, 1.793) | 0.219 |
| C4 (g/L) | 1.465 (0.283, 5.061) | 0.595 |
| FT4 (pmol/L | 0.007 (0.001, 0.028) | <0.001 |
| TgAb (uIU/ml) | 1.000 (1.000, 1.001) | 0.247 |
| TPOAb (uIU/ml) | 1.000 (0.996, 1.002) | 0.915 |

Anti-dsDNA, Anti-double stranded DNA Antibody; LYM, Lymphocytes; RBC-Blood, Red Blood Cells in Blood; HGB, Hemoglobin; ALB, Albumin; A/G, Albumin to Globulin Ratio; UA, Uric Acid; CO_2_-Blood, Carbon Dioxide in Blood; C3, Complement 3; FT3, Free Triiodothyronine; FT4, Free Thyroxine; TSH, Thyroid Stimulating Hormone; ANA, Antinuclear Antibody; Anti-Sm, Anti-Smith Antibody; Anti-SSA, Anti-Sjögren syndrome A Antibody; Anti-SSB, Anti-Sjögren syndrome B Antibody; Anti-RNP, Anti-ribonucleoprotein Antibody; Anti-Jo01, Anti-Jo-1 Antibody; WBC-Blood, White Blood Cells in Blood; NEU-Blood, Neutrophils in Blood; MONO, Monocytes; EOS, Eosinophils; BASO, Basophils; HCT, Hematocrit; MCV, Mean Corpuscular Volume; MCH, Mean Corpuscular Hemoglobin; MCHC, Mean Corpuscular Hemoglobin Concentration; RDW-SD, Red Cell Distribution Width-Standard Deviation; PLT, Platelets; MPV, Mean Platelet Volume; PCT-Blood, Platelet Count in Blood; PDW, Platelet Distribution Width; P-LCR, Platelet Large Cell Ratio; ALT, Alanine Aminotransferase; AST, Aspartate Aminotransferase; AST/ALT, Aspartate Aminotransferase to Alanine Aminotransferase Ratio; TP, Total Protein; GLO, Globulin; TBIL, Total Bilirubin; DBIL, Direct Bilirubin; IBIL, Indirect Bilirubin; TBA, Total Bile Acids; GGT, gamma-Glutamyltransferase; ALP-Blood, Alkaline Phosphatase in Blood; IgA, Immunoglobulin A; IgG, Immunoglobulin G; IgM, Immunoglobulin M; C4, Complement 4; TSH, Thyroid Stimulating Hormone; TgAb, Thyroglobulin Antibody; TPOAb, TPOAb, Thyroid Peroxidase Antibody.

Supplementary table 11 Multivariate logistic regression analysis of variables in SLE patients with ESS in Luzhou cohort.

| Variables | Coefficient β | OR (95% CI) | Z | P value |
| --- | --- | --- | --- | --- |
| UA (umol/L) | 0.003 | 1.003 (1.001, 1.005) | 2.673 | 0.008 |
| C3 (g/L) | -1.392 | 0.249 (0.050, 1.089) | -1.784 | 0.074 |
| FT3 (pmol/L) | -2.402 | 0.091 (0.035, 0.210) | -5.302 | <0.001 |
| TSH (uIU/ml) | -0.193 | 0.824 (0.676, 0.945) | -2.251 | 0.024 |

UA, Uric Acid; C3, Complement 3; FT3, Free Triiodothyronine; TSH, Thyroid Stimulating Hormone.

Supplementary table 12 Univariate logistic regression analysis of variables in SLE patients with clinical hyperthyroidism in Enshi cohort.

| Variables | OR (95% CI) | P value |
| --- | --- | --- |
| Sex | 3.761 (0.173, 40.050) | 0.283 |
| Age (year) | 1.045 (0.971, 1.131) | 0.238 |
| IgG (g/L) | 1.134 (1.044, 1.232) | 0.001 |
| FT4 (pmol/L) | 1.645 (1.206, 2.623) | 0.006 |
| Overlap syndrome (+) | 0.000 (NA, 3.115^125^) | 0.995 |
| WBC-Blood (10^9^/L) | 0.796 (0.423, 1.148) | 0.387 |
| NEU-Blood (10^9^/L) | 0.667 (0.262, 1.117) | 0.281 |
| LYM (10^9^/L) | 1.567 (0.294, 4.270) | 0.490 |
| MONO (10^9^/L) | 1.219 (0.009, 7.580) | 0.906 |
| EOS (10^9^/L) | 3.400 (0.000, 821.955) | 0.791 |
| BASO (10^9^/L) | 0.003 (0.000, 2.563^10^) | 0.872 |
| RBC-Blood (10^12^/L) | 2.000 (0.448, 9.322) | 0.397 |
| HGB (g/L) | 1.024 (0.976, 1.067) | 0.314 |
| HCT (%) | 1.000 (NA, 1.002) | 0.953 |
| MCV (fL) | 0.994 (0.876, 1.098) | 0.933 |
| MCH (pg) | 1.010 (0.745, 1.099) | 0.915 |
| MCHC (g/L) | 1.006 (0.952, 1.021) | 0.657 |
| RDW-SD (fL) | 0.900 (0.694, 1.038) | 0.375 |
| PLT (10^9^/L) | 1.003 (0.988, 1.017) | 0.647 |
| MPV (fL) | 0.675 (0.253, 1.537) | 0.389 |
| PCT-Blood (%) | 11.924 (0.000, 1.367^7^) | 0.751 |
| PDW (%) | 0.786 (0.475, 1.191) | 0.283 |
| P-LCR (%) | 0.939 (0.812, 1.050) | 0.323 |
| ALT (U/L) | 0.972 (0.817, 1.010) | 0.645 |
| AST (U/L) | 0.992 (0.860, 1.001) | 0.837 |
| AST/ALT | 0.911 (0.121, 1.641) | 0.884 |
| TP (g/L) | 1.112 (0.994, 1.274) | 0.097 |
| ALB (g/L) | 1.084 (0.925, 1.353) | 0.398 |
| GLO (g/L) | 1.114 (0.976, 1.259) | 0.082 |
| A/G | 0.349 (0.016, 5.643) | 0.485 |
| TBIL (umol/L) | 1.002 (NA, 1.023) | 0.919 |
| DBIL (umol/L) | 1.002 (NA, 1.034) | 0.970 |
| IBIL (umol/L) | 1.003 (NA, 1.049) | 0.941 |
| TBA (umol/L) | 0.891 (0.523, 1.028) | 0.578 |
| GGT (U/L) | 0.990 (0.924, 1.007) | 0.672 |
| ALP-Blood (U/L) | 0.987 (0.933, 1.005) | 0.575 |
| Urea-Blood (mmol/L) | 0.925 (0.617, 1.092) | 0.611 |
| UA (umol/L) | 0.996 (0.983, 1.005) | 0.488 |
| Crea-Blood (U/L) | 1.007 (0.942, 1.062) | 0.810 |
| K (mmol/L) | 2.032 (0.358, 6.087) | 0.288 |
| Cl (mmol/L) | 0.997 (0.973, 1.031) | 0.828 |
| Ca (mmol/L) | 1.001 (0.966, 1.027) | 0.928 |
| CO_2_-Blood (mmol/L) | 0.970 (0.774, 1.333) | 0.822 |
| IgA (g/L) | 0.737 (0.205, 1.756) | 0.617 |
| IgM (g/L) | 0.830 (0.067, 3.564) | 0.857 |
| C3 (g/L) | 0.798 (0.011, 80.621) | 0.920 |
| C4 (g/L) | 0.008 (0.000, 656.151) | 0.469 |
| FT3 (pmol/L) | 14.746 (2.710, 770.005) | 0.023 |
| TSH (uIU/ml) | 0.000 (0.000, 0.000) | 0.252 |

IgG, Immunoglobulin G; FT4, Free Thyroxine; WBC-Blood, White Blood Cells in Blood; NEU-Blood, Neutrophils in Blood; LYM, Lymphocytes; MONO, Monocytes; EOS, Eosinophils; BASO, Basophils; RBC-Blood, Red Blood Cells in Blood; HGB, Hemoglobin; HCT, Hematocrit; MCV, Mean Corpuscular Volume; MCH, Mean Corpuscular Hemoglobin; MCHC, Mean Corpuscular Hemoglobin Concentration; RDW-SD, Red Cell Distribution Width-Standard Deviation; PLT, Platelets; MPV, Mean Platelet Volume; PCT-Blood, Platelet Count in Blood; PDW, Platelet Distribution Width; P-LCR, Platelet Large Cell Ratio; ALT, Alanine Aminotransferase; AST, Aspartate Aminotransferase; AST/ALT, Aspartate Aminotransferase to Alanine Aminotransferase Ratio; TP, Total Protein; ALB, Albumin; GLO, Globulin; A/G, Albumin to Globulin Ratio; TBIL, Total Bilirubin; DBIL, Direct Bilirubin; IBIL, Indirect Bilirubin; TBA, Total Bile Acids; GGT, gamma-Glutamyltransferase; ALP-Blood, Alkaline Phosphatase in Blood; UA, Uric Acid; CO_2_-Blood, Carbon Dioxide in Blood; IgA, Immunoglobulin A; IgM, Immunoglobulin M; C3, Complement 3; C4, Complement 4; FT3, Free Triiodothyronine; TSH, Thyroid Stimulating Hormone.

Supplementary table 13 Multivariate logistic regression analysis of variables in SLE patients with clinical hyperthyroidism in Enshi cohort.

| Variables | Coefficient β | OR (95% CI) | Z | P value |
| --- | --- | --- | --- | --- |
| Age (year) | 0.111 | 1.117 (1.002, 1.290) | 1.883 | 0.060 |
| FT4 (pmol/L) | 0.663 | 1.941 (1.286, 4.227) | 2.478 | 0.013 |

FT4, Free Thyroxine.

Supplementary table 14 Univariate logistic regression analysis of variables in SLE patients with subclinical hyperthyroidism in Enshi cohort.

| Variables | OR (95% CI) | P value |
| --- | --- | --- |
| Sex | 2.477 (0.777, 6.716) | 0.093 |
| Age (year) | 0.986 (0.958, 1.015) | 0.339 |
| LYM (10^9^/L) | 0.274 (0.103, 0.652) | 0.006 |
| MCV (fL) | 1.050 (1.002, 1.105) | 0.035 |
| RDW-SD (fL) | 1.045 (1.013, 1.094) | 0.040 |
| Ca (mmol/L) | 0.979 (0.952, 0.996) | 0.049 |
| Overlap syndrome (+) | 0.694 (0.038, 3.561) | 0.727 |
| WBC-Blood (10^9^/L) | 1.033 (0.918, 1.132) | 0.542 |
| NEU-Blood (10^9^/L) | 1.070 (0.958, 1.170) | 0.172 |
| MONO (10^9^/L) | 0.184 (0.018, 1.272) | 0.125 |
| EOS (10^9^/L) | 0.000 (0.000, 0.000) | 0.001 |
| BASO (10^9^/L) | 0.000 (0.000, 0.097) | 0.068 |
| RBC-Blood (10^12^/L) | 0.729 (0.434, 1.266) | 0.243 |
| HGB (g/L) | 0.997 (0.979, 1.016) | 0.768 |
| HCT (%) | 0.979 (0.921, 1.000) | 0.513 |
| MCH (pg) | 1.075 (1.012, 1.231) | 0.092 |
| MCHC (g/L) | 1.013 (1.001, 1.034) | 0.062 |
| PLT (10^9^/L) | 0.996 (0.990, 1.002) | 0.232 |
| MPV (fL) | 1.060 (0.776, 1.430) | 0.707 |
| PCT-Blood (%) | 0.082 (0.000, 39.435) | 0.438 |
| PDW (%) | 1.025 (0.876, 1.195) | 0.754 |
| P-LCR (%) | 1.008 (0.968, 1.048) | 0.699 |
| ALT (U/L) | 0.998 (0.976, 1.004) | 0.772 |
| AST (U/L) | 1.000 (NA, 1.003) | 0.968 |
| AST/ALT | 0.881 (0.460, 1.272) | 0.625 |
| TP (g/L) | 1.003 (0.975, 1.037) | 0.871 |
| ALB (g/L) | 0.992 (0.940, 1.052) | 0.774 |
| GLO (g/L) | 0.993 (0.931, 1.054) | 0.834 |
| A/G | 1.543 (0.522, 4.309) | 0.420 |
| TBIL (umol/L) | 0.997 (0.946, 1.014) | 0.861 |
| DBIL (umol/L) | 1.001 (NA, 1.023) | 0.945 |
| IBIL (umol/L) | 0.983 (0.866, 1.024) | 0.651 |
| TBA (umol/L) | 1.006 (0.974, 1.023) | 0.521 |
| GGT (U/L) | 0.999 (0.987, 1.004) | 0.790 |
| ALP-Blood (U/L) | 0.997 (0.984, 1.003) | 0.575 |
| Urea-Blood (mmol/L) | 1.036 (0.982, 1.081) | 0.136 |
| UA (umol/L) | 1.002 (0.998, 1.005) | 0.324 |
| Crea-Blood (U/L) | 0.977 (0.947, 1.002) | 0.103 |
| K (mmol/L) | 1.283 (0.596, 2.412) | 0.480 |
| Cl (mmol/L) | 1.016 (1.001, 1.037) | 0.068 |
| CO_2_-Blood (mmol/L) | 0.979 (0.886, 1.095) | 0.687 |
| IgA (g/L) | 1.113 (0.760, 1.506) | 0.535 |
| IgG (g/L) | 1.009 (0.932, 1.071) | 0.794 |
| IgM (g/L) | 1.020 (0.462, 1.928) | 0.957 |
| C3 (g/L) | 0.742 (0.137, 4.220) | 0.732 |
| C4 (g/L) | 4.126 (0.055, 199.034) | 0.496 |
| FT3 (pmol/L) | 0.753 (0.519, 1.074) | 0.140 |
| FT4 (pmol/L) | 1.053 (0.964, 1.137) | 0.183 |
| TSH (uIU/ml) | 0.001 (0.000, 0.015) | <0.001 |

LYM, Lymphocytes; MCV, Mean Corpuscular Volume; RDW-SD, Red Cell Distribution Width-Standard Deviation; WBC-Blood, White Blood Cells in Blood; NEU-Blood, Neutrophils in Blood; MONO, Monocytes; EOS, Eosinophils; BASO, Basophils; RBC-Blood, Red Blood Cells in Blood; HGB, Hemoglobin; HCT, Hematocrit; MCH, Mean Corpuscular Hemoglobin; MCHC, Mean Corpuscular Hemoglobin Concentration; PLT, Platelets; MPV, Mean Platelet Volume; PCT-Blood, Platelet Count in Blood; PDW, Platelet Distribution Width; P-LCR, Platelet Large Cell Ratio; ALT, Alanine Aminotransferase; AST, Aspartate Aminotransferase; AST/ALT, Aspartate Aminotransferase to Alanine Aminotransferase Ratio; TP, Total Protein; ALB, Albumin; GLO, Globulin; A/G, Albumin to Globulin Ratio; TBIL, Total Bilirubin; DBIL, Direct Bilirubin; IBIL, Indirect Bilirubin; TBA, Total Bile Acids; GGT, gamma-Glutamyltransferase; ALP-Blood, Alkaline Phosphatase in Blood; UA, Uric Acid; CO_2_-Blood, Carbon Dioxide in Blood; IgA, Immunoglobulin A; IgG, Immunoglobulin G; IgM, Immunoglobulin M; C3, Complement 3; C4, Complement 4; FT3, Free Triiodothyronine; FT4, Free Thyroxine; TSH, Thyroid Stimulating Hormone.

Supplementary table 15 Multivariate logistic regression analysis of variables in SLE patients with subclinical hyperthyroidism in Enshi cohort.

| Variables | Coefficient β | OR (95% CI) | Z | P value |
| --- | --- | --- | --- | --- |
| Age (year) | -0.021 | 0.979 (0.951, 1.008) | -1.428 | 0.153 |
| LYM (10^9^/L) | -1.000 | 0.368 (0.137, 0.862) | -2.131 | 0.033 |
| RDW-SD (fL) | 0.034 | 1.035 (1.008, 1.081) | 1.961 | 0.049 |
| Ca (mmol/L) | -0.016 | 0.984 (0.957, 1.001) | -1.493 | 0.135 |

LYM, Lymphocytes; RDW-SD, Red Cell Distribution Width-Standard Deviation.

Supplementary table 16 Univariate logistic regression analysis of variables in SLE patients with clinical hypothyroidism in Enshi cohort.

| Variables | OR (95% CI) | P value |
| --- | --- | --- |
| Sex | 1.758 (0.392, 5.731) | 0.393 |
| Age (year) | 1.004 (0.972, 1.037) | 0.813 |
| WBC-Blood (10^9^/L) | 0.764 (0.586, 0.945) | 0.028 |
| NEU-Blood (10^9^/L) | 0.729 (0.526, 0.937) | 0.032 |
| MONO (10^9^/L) | 0.045 (0.002, 0.585) | 0.027 |
| GGT (U/L) | 1.005 (1.001, 1.010) | 0.030 |
| FT3 (pmol/L) | 0.591 (0.391, 0.900) | 0.013 |
| FT4 (pmol/L) | 0.554 (0.430, 0.675) | <0.001 |
| TSH (uIU/ml) | 1.297 (1.175, 1.445) | <0.001 |
| Overlap syndrome (+) | 0.939 (0.051, 4.947) | 0.952 |
| LYM (10^9^/L) | 0.838 (0.350, 1.726) | 0.668 |
| EOS (10^9^/L) | 2.184 (0.004, 78.801) | 0.736 |
| BASO (10^9^/L) | 0.000 (0.000, 965.447) | 0.209 |
| RBC-Blood (10^12^/L) | 1.201 (0.632, 2.411) | 0.594 |
| HGB (g/L) | 1.003 (0.982, 1.025) | 0.817 |
| HCT (%) | 0.999 (NA, 1.001) | 0.883 |
| MCV (fL) | 0.979 (0.923, 1.040) | 0.506 |
| MCH (pg) | 0.951 (0.818, 1.057) | 0.535 |
| MCHC (g/L) | 0.995 (0.963, 1.013) | 0.767 |
| RDW-SD (fL) | 0.994 (0.918, 1.028) | 0.830 |
| PLT (10^9^/L) | 0.994 (0.986, 1.001) | 0.083 |
| MPV (fL) | 1.191 (0.843, 1.665) | 0.311 |
| PCT-Blood (%) | 0.000 (0.000, 0.727) | 0.046 |
| PDW (%) | 1.056 (0.884, 1.255) | 0.540 |
| P-LCR (%) | 1.022 (0.977, 1.068) | 0.326 |
| ALT (U/L) | 0.997 (0.967, 1.005) | 0.759 |
| AST (U/L) | 0.999 (NA, 1.003) | 0.819 |
| AST/ALT | 1.065 (0.637, 1.420) | 0.739 |
| TP (g/L) | 1.012 (0.979, 1.056) | 0.531 |
| ALB (g/L) | 0.986 (0.928, 1.053) | 0.655 |
| GLO (g/L) | 1.034 (0.967, 1.099) | 0.309 |
| A/G | 0.331 (0.087, 1.202) | 0.100 |
| TBIL (umol/L) | 0.976 (0.873, 1.013) | 0.573 |
| DBIL (umol/L) | 0.933 (0.679, 1.024) | 0.578 |
| IBIL (umol/L) | 0.959 (0.795, 1.024) | 0.573 |
| TBA (umol/L) | 1.002 (0.939, 1.021) | 0.922 |
| ALP-Blood (U/L) | 1.002 (0.996, 1.005) | 0.356 |
| Urea-Blood (mmol/L) | 1.014 (0.936, 1.069) | 0.665 |
| UA (umol/L) | 1.003 (0.999, 1.006) | 0.093 |
| Crea-Blood (U/L) | 1.013 (0.988, 1.037) | 0.292 |
| K (mmol/L) | 1.226 (0.499, 2.497) | 0.618 |
| Cl (mmol/L) | 0.993 (0.983, 1.005) | 0.223 |
| Ca (mmol/L) | 1.007 (0.996, 1.018) | 0.180 |
| CO_2_-Blood (mmol/L) | 0.952 (0.856, 1.076) | 0.400 |
| IgA (g/L) | 1.188 (0.791, 1.626) | 0.337 |
| IgG (g/L) | 1.000 (0.907, 1.072) | 0.999 |
| IgM (g/L) | 1.626 (0.798, 2.943) | 0.133 |
| C3 (g/L) | 0.565 (0.084, 4.034) | 0.562 |
| C4 (g/L) | 0.022 (0.000, 4.442) | 0.185 |

WBC-Blood, White Blood Cells in Blood; NEU-Blood, Neutrophils in Blood; MONO, Monocytes; GGT, gamma-Glutamyltransferase; FT3, Free Triiodothyronine; FT4, Free Thyroxine; TSH, Thyroid Stimulating Hormone; LYM, Lymphocytes; EOS, Eosinophils; BASO, Basophils; RBC-Blood, Red Blood Cells in Blood; HGB, Hemoglobin; HCT, Hematocrit; MCV, Mean Corpuscular Volume; MCH, Mean Corpuscular Hemoglobin; MCHC, Mean Corpuscular Hemoglobin Concentration; RDW-SD, Red Cell Distribution Width-Standard Deviation; PLT, Platelets; MPV, Mean Platelet Volume; PCT-Blood, Platelet Count in Blood; PDW, Platelet Distribution Width; P-LCR, Platelet Large Cell Ratio; ALT, Alanine Aminotransferase; AST, Aspartate Aminotransferase; AST/ALT, Aspartate Aminotransferase to Alanine Aminotransferase Ratio; TP, Total Protein; ALB, Albumin; GLO, Globulin; A/G, Albumin to Globulin Ratio; TBIL, Total Bilirubin; DBIL, Direct Bilirubin; IBIL, Indirect Bilirubin; TBA, Total Bile Acids; ALP-Blood, Alkaline Phosphatase in Blood; UA, Uric Acid; CO_2_-Blood, Carbon Dioxide in Blood; IgA, Immunoglobulin A; IgG, Immunoglobulin G; IgM, Immunoglobulin M; C3, Complement 3; C4, Complement 4.

Supplementary table 17 Multivariate logistic regression analysis of variables in SLE patients with clinical hypothyroidism in Enshi cohort.

| Variables | Coefficient β | OR (95% CI) | Z | P value |
| --- | --- | --- | --- | --- |
| Age (year) | 0.059 | 1.060 (0.992, 1.138) | 1.732 | 0.083 |
| GGT (U/L) | -0.046 | 0.955 (0.912, 0.984) | -2.350 | 0.019 |
| FT3 (pmol/L) | 0.754 | 2.125 (0.814, 6.921) | 1.403 | 0.161 |
| FT4 (pmol/L) | -1.133 | 0.322 (0.150, 0.523) | -3.688 | <0.001 |
| TSH (uIU/ml) | 0.433 | 1.542 (1.283, 1.991) | 4.004 | <0.001 |

GGT, gamma-Glutamyltransferase; FT3, Free Triiodothyronine; FT4, Free Thyroxine; TSH, Thyroid Stimulating Hormone.

Supplementary table 18 Univariate logistic regression analysis of variables in SLE patients with subclinical hypothyroidism in Enshi cohort.

| Variables | OR (95% CI) | P value |
| --- | --- | --- |
| Sex | 0.289 (0.069, 0.826) | 0.043 |
| Age (year) | 1.012 (0.995, 1.030) | 0.155 |
| GLO (g/L) | 1.049 (1.014, 1.087) | 0.006 |
| A/G | 0.366 (0.180, 0.725) | 0.005 |
| IgA (g/L) | 1.292 (1.050, 1.598) | 0.015 |
| IgM (g/L) | 1.616 (1.093, 2.384) | 0.015 |
| TSH (uIU/ml) | 1.038 (1.011, 1.090) | 0.041 |
| Overlap syndrome (+) | 2.253 (0.885, 5.312) | 0.072 |
| WBC-Blood (10^9^/L) | 1.031 (0.963, 1.097) | 0.354 |
| NEU-Blood (10^9^/L) | 1.025 (0.952, 1.097) | 0.482 |
| LYM (10^9^/L) | 1.079 (0.724, 1.568) | 0.699 |
| MONO (10^9^/L) | 1.443 (0.627, 3.131) | 0.352 |
| EOS (10^9^/L) | 129.45 (9.617, 2432.543) | 0.001 |
| BASO (10^9^/L) | 169.067 (0.002, 6.141^6^) | 0.321 |
| RBC-Blood (10^12^/L) | 0.966 (0.693, 1.359) | 0.838 |
| HGB (g/L) | 0.994 (0.983, 1.005) | 0.308 |
| HCT (%) | 0.980 (0.943, 1.001) | 0.283 |
| MCV (fL) | 0.974 (0.942, 1.007) | 0.127 |
| MCH (pg) | 0.938 (0.861, 1.014) | 0.147 |
| MCHC (g/L) | 0.994 (0.977, 1.007) | 0.452 |
| RDW-SD (fL) | 1.003 (0.975, 1.024) | 0.790 |
| PLT (10^9^/L) | 1.000 (0.997, 1.004) | 0.783 |
| MPV (fL) | 0.980 (0.814, 1.175) | 0.828 |
| PCT-Blood (%) | 0.600 (0.015, 22.676) | 0.785 |
| PDW (%) | 1.023 (0.932, 1.122) | 0.625 |
| P-LCR (%) | 0.999 (0.976, 1.023) | 0.966 |
| ALT (U/L) | 1.001 (0.996, 1.004) | 0.595 |
| AST (U/L) | 1.001 (0.998, 1.003) | 0.580 |
| AST/ALT | 0.993 (0.759, 1.223) | 0.951 |
| TP (g/L) | 1.012 ( 0.994 , 1.033 ) | 0.223 |
| ALB (g/L) | 0.986 (0.954, 1.020) | 0.397 |
| TBIL (umol/L) | 0.998 (0.975, 1.010) | 0.783 |
| DBIL (umol/L) | 0.995 (0.934, 1.016) | 0.743 |
| IBIL (umol/L) | 0.997 (0.962, 1.019) | 0.815 |
| TBA (umol/L) | 0.984 (0.932, 1.009) | 0.452 |
| GGT (U/L) | 1.003 (1.000, 1.006) | 0.113 |
| ALP-Blood (U/L) | 1.003 (1.000, 1.007) | 0.052 |
| Urea-Blood (mmol/L) | 0.993 (0.950, 1.031) | 0.745 |
| UA (umol/L) | 1.002 (1.000, 1.004) | 0.083 |
| Crea-Blood (U/L) | 1.008 (0.994, 1.020) | 0.256 |
| K (mmol/L) | 0.854 (0.515, 1.352) | 0.520 |
| Cl (mmol/L) | 0.997 (0.991, 1.004) | 0.399 |
| Ca (mmol/L) | 1.003 (0.996, 1.009) | 0.394 |
| CO_2_-Blood (mmol/L) | 1.054 (0.985, 1.134) | 0.146 |
| IgG (g/L) | 1.040 (1.000, 1.082) | 0.046 |
| C3 (g/L) | 0.879 (0.319, 2.456) | 0.804 |
| C4 (g/L) | 0.387 (0.025, 5.088) | 0.482 |
| FT3 (pmol/L) | 1.051 (0.896, 1.218) | 0.483 |
| FT4 (pmol/L) | 1.014 (0.953, 1.074) | 0.633 |

GLO, Globulin; A/G, Albumin to Globulin Ratio; IgA, Immunoglobulin A; IgM, Immunoglobulin M; TSH, Thyroid Stimulating Hormone; WBC-Blood, White Blood Cells in Blood; NEU-Blood, Neutrophils in Blood; LYM, Lymphocytes; MONO, Monocytes; EOS, Eosinophils; BASO, Basophils; RBC-Blood, Red Blood Cells in Blood; HGB, Hemoglobin; HCT, Hematocrit; MCV, Mean Corpuscular Volume; MCH, Mean Corpuscular Hemoglobin; MCHC, Mean Corpuscular Hemoglobin Concentration; RDW-SD, Red Cell Distribution Width-Standard Deviation; PLT, Platelets; MPV, Mean Platelet Volume; PCT-Blood, Platelet Count in Blood; PDW, Platelet Distribution Width; P-LCR, Platelet Large Cell Ratio; ALT, Alanine Aminotransferase; AST, Aspartate Aminotransferase; AST/ALT, Aspartate Aminotransferase to Alanine Aminotransferase Ratio; TP, Total Protein; ALB, Albumin; TBIL, Total Bilirubin; DBIL, Direct Bilirubin; IBIL, Indirect Bilirubin; TBA, Total Bile Acids; GGT, gamma-Glutamyltransferase; ALP-Blood, Alkaline Phosphatase in Blood; UA, Uric Acid; CO_2_-Blood, Carbon Dioxide in Blood; IgG, Immunoglobulin G; C3, Complement 3; C4, Complement 4; FT3, Free Triiodothyronine; FT4, Free Thyroxine.

Supplementary table 19 Multivariate logistic regression analysis of variables in SLE patients with subclinical hypothyroidism in Enshi cohort.

| Variables | Coefficient β | OR (95% CI) | Z | P value |
| --- | --- | --- | --- | --- |
| Sex | -1.391 | 0.249 (0.050, 0.779) | -2.065 | 0.039 |
| A/G | -0.765 | 0.465 (0.222, 0.950) | -2.066 | 0.039 |
| IgM (g/L) | 0.328 | 1.388 (0.914, 2.094) | 1.570 | 0.116 |
| TSH (uIU/ml) | 0.036 | 1.037 (1.009, 1.078) | 2.271 | 0.023 |

A/G, Albumin to Globulin Ratio; IgM, Immunoglobulin M; TSH, Thyroid Stimulating Hormone.

Supplementary table 20 Univariate logistic regression analysis of variables in SLE patients with ESS in Enshi cohort.

| Variables | OR (95% CI) | P value |
| --- | --- | --- |
| Sex | 0.376 (0.021, 1.881) | 0.346 |
| Age (year) | 0.977 (0.948, 1.006) | 0.128 |
| MONO (10^9^/L) | 0.062 (0.005, 0.596) | 0.025 |
| RBC-Blood (10^12^/L) | 0.522 (0.312, 0.886) | 0.014 |
| HGB (g/L) | 0.977 (0.959, 0.994) | 0.009 |
| HCT (%) | 0.923 (0.869, 0.984) | 0.009 |
| ALB (g/L) | 0.924 (0.877, 0.974) | 0.003 |
| A/G | 0.149 (0.042, 0.499) | 0.002 |
| TBIL (umol/L) | 1.015 (1.002, 1.032) | 0.035 |
| TBA (umol/L) | 1.019 (1.002, 1.042) | 0.036 |
| Urea-Blood (mmol/L) | 1.075 (1.031, 1.121) | 0.001 |
| UA (umol/L) | 1.005 (1.002, 1.008) | 0.001 |
| K (mmol/L) | 2.400 (1.323, 4.227) | 0.002 |
| CO_2_-Blood (mmol/L) | 0.845 (0.771, 0.926) | <0.001 |
| C3 (g/L) | 0.087 (0.015, 0.483) | 0.005 |
| FT3 (pmol/L) | 0.272 (0.166, 0.417) | <0.001 |
| FT4 (pmol/L) | 0.585 (0.472, 0.696) | <0.001 |
| Overlap syndrome (+) | 0.732 (0.040, 3.775) | 0.766 |
| WBC-Blood (10^9^/L) | 0.939 (0.795, 1.065) | 0.397 |
| NEU-Blood (10^9^/L) | 0.975 (0.827, 1.099) | 0.729 |
| LYM (10^9^/L) | 0.466 (0.190, 1.027) | 0.077 |
| EOS (10^9^/L) | 0.000 (0.000, 0.000) | 0.006 |
| BASO (10^9^/L) | 0.000 (0.000, 0.000) | 0.008 |
| MCV (fL) | 1.000 (0.946, 1.053) | 0.991 |
| MCH (pg) | 0.985 (0.858, 1.061) | 0.811 |
| MCHC (g/L) | 0.995 (0.966, 1.012) | 0.705 |
| RDW-SD (fL) | 1.019 (0.991, 1.047) | 0.108 |
| PLT (10^9^/L) | 0.997 (0.991, 1.003) | 0.396 |
| MPV (fL) | 0.865 (0.616, 1.190) | 0.387 |
| PCT-Blood (%) | 0.021 (0.000, 12.719) | 0.248 |
| PDW (%) | 1.068 (0.910, 1.248) | 0.415 |
| P-LCR (%) | 0.980 (0.936, 1.022) | 0.352 |
| ALT (U/L) | 1.003 (0.998, 1.007) | 0.125 |
| AST (U/L) | 1.002 (0.999, 1.004) | 0.131 |
| AST/ALT | 1.037 (0.639, 1.374) | 0.844 |
| TP (g/L) | 0.992 (0.967, 1.023) | 0.551 |
| GLO (g/L) | 1.037 (0.976, 1.096) | 0.219 |
| DBIL (umol/L) | 1.050 (1.010, 1.120) | 0.094 |
| IBIL (umol/L) | 1.015 (0.981, 1.038) | 0.242 |
| GGT (U/L) | 1.000 (0.990, 1.004) | 0.967 |
| ALP-Blood (U/L) | 1.002 (0.997, 1.005) | 0.256 |
| Crea-Blood (U/L) | 1.011 (0.989, 1.033) | 0.310 |
| Cl (mmol/L) | 0.999 (0.989, 1.011) | 0.913 |
| Ca (mmol/L) | 1.002 (0.991, 1.012) | 0.663 |
| IgA (g/L) | 0.678 (0.410, 1.067) | 0.116 |
| IgG (g/L) | 0.989 (0.903, 1.059) | 0.789 |
| IgM (g/L) | 1.009 (0.446, 1.938) | 0.981 |
| C4 (g/L) | 0.962 (0.009, 62.786) | 0.986 |
| TSH (uIU/ml) | 0.873 (0.692, 1.008) | 0.182 |

ESS, Euthyroid Sick Syndrome; MONO, Monocytes; RBC-Blood, Red Blood Cells in Blood; HGB, Hemoglobin; HCT, Hematocrit; ALB, Albumin; A/G, Albumin to Globulin Ratio; TBIL, Total Bilirubin; TBA, Total Bile Acids; UA, Uric Acid; CO_2_-Blood, Carbon Dioxide in Blood; C3, Complement 3; FT3, Free Triiodothyronine; FT4, Free Thyroxine; WBC-Blood, White Blood Cells in Blood; NEU-Blood, Neutrophils in Blood; LYM, Lymphocytes; EOS, Eosinophils; BASO, Basophils; MCV, Mean Corpuscular Volume; MCH, Mean Corpuscular Hemoglobin; MCHC, Mean Corpuscular Hemoglobin Concentration; RDW-SD, Red Cell Distribution Width-Standard Deviation; PLT, Platelets; MPV, Mean Platelet Volume; PCT-Blood, Platelet Count in Blood; PDW, Platelet Distribution Width; P-LCR, Platelet Large Cell Ratio; ALT, Alanine Aminotransferase; AST, Aspartate Aminotransferase; AST/ALT, Aspartate Aminotransferase to Alanine Aminotransferase Ratio; TP, Total Protein; GLO, Globulin; DBIL, Direct Bilirubin; IBIL, Indirect Bilirubin; GGT, gamma-Glutamyltransferase; ALP-Blood, Alkaline Phosphatase in Blood; IgA, Immunoglobulin A; IgG, Immunoglobulin G; IgM, Immunoglobulin M; C4, Complement 4; TSH, Thyroid Stimulating Hormone.

Supplementary table 21 Multivariate logistic regression analysis of variables in SLE patients with ESS in Enshi cohort.

| Variables | Coefficient β | OR (95% CI) | Z | P value |
| --- | --- | --- | --- | --- |
| Age (year) | -0.032 | 0.968 (0.929, 1.006) | -1.617 | 0.106 |
| TBIL (umol/L) | 0.064 | 1.066 (1.004, 1.124) | 2.049 | 0.040 |
| TBA (umol/L) | -0.067 | 0.935 (0.870, 1.009) | -1.619 | 0.105 |
| C3 (g/L) | -2.084 | 0.124 (0.014, 0.971) | -1.959 | 0.049 |
| FT3 (pmol/L) | -0.770 | 0.463 (0.254, 0.806) | -2.640 | 0.008 |
| FT4 (pmol/L) | -0.448 | 0.639 (0.502, 0.784) | -3.968 | <0.001 |

TBIL, Total Bilirubin; TBA, Total Bile Acids; C3, Complement 3; FT3, Free Triiodothyronine; FT4, Free Thyroxine.

Supplementary table 22 Results of meta-analysis of factors influencing thyroid dysfunction in patients with SLE.

| Factors | No. of studies included | Heterogeneity test | | Effects model | Meta-analysis results | |
| --- | --- | --- | --- | --- | --- | --- |
|  |  | I^2^ (%) | P value |  | OR (95%CI) | P value |
| Clinical hypothyroidism |  |  |  |  |  |  |
| Sex | 5 | 87.8 | <0.001 | Random effects model | 0.763 (0.312, 1.866) | 0.553 |
| Age (year) | 4 | 0.0 | 0.431 | Fixed effects model | 0.290 (-2.399, 2.980) | 0.832 |
| Rash (+) | 2 | 81.9 | 0.0188 | Random effects model | 1.398 (0.112, 17.428) | 0.794 |
| Arthritis (+) | 2 | 2.7 | 0.311 | Fixed effects model | 1.440 (0.740, 2.802) | 0.283 |
| Renal involvement (+) | 3 | 66.6 | 0.049 | Random effects model | 2.761 (1.202, 6.341) | 0.017 |
| Anti-dsDNA (+) | 5 | 39.5 | 0.115 | Fixed effects model | 1.432 (0.917, 2.237) | 0.115 |
| Oral ulcer (+) | 2 | 0.0 | 0.799 | Fixed effects model | 0.671 (0.300, 1.502) | 0.332 |
| ALB (g/L) | 2 | 99.3 | <0.001 | Random effects model | 1.941 (-13.709, 17.592) | 0.808 |
| TG (mg/dl) | 2 | 97.9 | <0.001 | Random effects model | 2.313 (-0.481, 5.107) | 0.105 |
| ESR (mm/h) | 2 | 69.2 | 0.072 | Random effects model | 6.806 (-2.995, 16.607) | 0.174 |
| Anti-Sm (+) | 4 | 0.0 | 0.888 | Fixed effects model | 0.716 (0.474, 1.082) | 0.113 |
| Anti-RNP (+) | 2 | 0.0 | 0.926 | Fixed effects model | 0.997 (0.395, 2.520) | 0.9956 |
| Anti-SSA (+) | 3 | 0.0 | 0.763 | Fixed effects model | 1.199 (0.749, 1.920) | 0.449 |
| Anti-SSB (+) | 3 | 0.0 | 0.791 | Fixed effects model | 0.708 (0.322, 1.556) | 0.389 |
| FT3 (pmol/L) | 2 | 0.0 | 0.832 | Random effects model | -0.547 (-0.709, -0.385) | <0.001 |
| TSH (uIU/ml) | 2 | 86.5 | 0.007 | Random effects model | 16.091 (-0.311, 32.493) | 0.055 |
| ESS |  |  |  |  |  |  |
| C3 (g/L) | 2 | 0.0 | 0.334 | Fixed Effects Model | -0.231 (-0.311, -0.151) | <0.001 |
| FT3 (pmol/L) | 2 | 96.0 | <0.001 | Random effects model | -1.263 (-2.173, -0.353) | 0.007 |

Anti-dsDNA, Anti-double stranded DNA Antibody; ALB, Albumin; TG, Triglycerides; ESR, Erythrocyte Sedimentation Rate; Anti-Sm, Anti-Smith Antibody; Anti-RNP, Anti-ribonucleoprotein Antibody; Anti-SSA, Anti-Sjögren syndrome A Antibody; Anti-SSB, Anti-Sjögren syndrome B Antibody; FT3, Free Triiodothyronine; TSH, Thyroid Stimulating Hormone; ESS, Euthyroid Sick Syndrome; C3, Complement 3.
